# Global analysis of putative phospholipases in *Plasmodium falciparum* reveals an essential role of the phosphoinositide-specific phospholipase C in parasite maturation

**DOI:** 10.1128/mbio.01413-23

**Published:** 2023-07-25

**Authors:** Paul-Christian Burda, Abhinay Ramaprasad, Sabrina Bielfeld, Emma Pietsch, Anna Woitalla, Christoph Söhnchen, Mehar Nihal Singh, Jan Strauss, Aaron Sait, Lucy M. Collinson, Dominik Schwudke, Michael J. Blackman, Tim-Wolf Gilberger

**Affiliations:** 1 Centre for Structural Systems Biology, Hamburg, Germany; 2 Bernhard Nocht Institute for Tropical Medicine, Hamburg, Germany; 3 University of Hamburg, Hamburg, Germany; 4 Malaria Biochemistry Laboratory, The Francis Crick Institute, London, United Kingdom; 5 Division of Bioanalytical Chemistry, Research Center Borstel, Leibniz Lung Center, Borstel, Germany; 6 Division of Infection and Immunity, University College London, London, United Kingdom; 7 Electron Microscopy Science Technology Platform, The Francis Crick Institute, London, United Kingdom; 8 German Center for Infection Research, Thematic Translational Unit Tuberculosis, Partner Site Hamburg-Lübeck-Borstel-Riems, Borstel, Germany; 9 German Center for Lung Research (DZL), Airway Research Center North (ARCN), Research Center Borstel, Leibniz Lung Center, Borstel, Germany; 10 Faculty of Infectious and Tropical Diseases, London School of Hygiene & Tropical Medicine, London, United Kingdom; NIAID/NIH, Rockville, Maryland, USA

**Keywords:** malaria, blood stage, phospholipase, phosphoinositides

## Abstract

For its replication within red blood cells, the malaria parasite depends on a highly active and regulated lipid metabolism. Enzymes involved in lipid metabolic processes such as phospholipases are, therefore, potential drug targets. Here, using reverse genetics approaches, we show that only 1 out of the 19 putative phospholipases expressed in asexual blood stages of *Plasmodium falciparum* is essential for proliferation *in vitro*, pointing toward a high level of redundancy among members of this enzyme family. Using conditional mislocalization and gene disruption techniques, we show that this essential phosphoinositide-specific phospholipase C (PI-PLC, PF3D7_1013500) has a previously unrecognized essential role during intracellular parasite maturation, long before its previously perceived role in parasite egress and invasion. Subsequent lipidomic analysis suggests that PI-PLC mediates cleavage of phosphatidylinositol bisphosphate (PIP_2_) in schizont-stage parasites, underlining its critical role in regulating phosphoinositide levels in the parasite.

**IMPORTANCE:**

The clinical symptoms of malaria arise due to repeated rounds of replication of *Plasmodium* parasites within red blood cells (RBCs). Central to this is an intense period of membrane biogenesis. Generation of membranes not only requires *de novo* synthesis and acquisition but also the degradation of phospholipids, a function that is performed by phospholipases. In this study, we investigate the essentiality of the 19 putative phospholipase enzymes that the human malaria parasite *Plasmodium falciparum* expresses during its replication within RBCs. We not only show that a high level of functional redundancy exists among these enzymes but, at the same time, also identify an essential role for the phosphoinositide-specific phospholipase C in parasite development and cleavage of the phospholipid phosphatidylinositol bisphosphate.

## INTRODUCTION

With an estimated 247 million cases per year worldwide and more than 600,000 deaths, malaria remains one of the most important human health threats (1). The replication of protozoan parasites of the genus *Plasmodium* within red blood cells (RBCs) and the associated transformation and destruction of these cells are responsible for the clinical symptoms of the disease. With no effective vaccine widely available and widespread resistance of the parasite to available drugs, there is an urgent need to better understand its cell biology to find new ways of combating this important pathogen.

Malaria parasites are transmitted by the bite of an infected *Anophele*s mosquito and initially establish in their vertebrate host by multiplying in hepatocytes. From here, parasites are released into the bloodstream, where they undergo repeated cycles of replication within RBCs (2). Central to intraerythrocytic growth of parasites is an intense period of membrane biogenesis. Not only do the intracellular parasites need to extend the parasite plasma membrane (PPM) and the parasitophorous vacuole membrane (PVM), which surrounds them during their multiplication. During the formation of daughter cells, they also have to replicate and synthesize their organelles and the inner membrane complex, which are flattened membranous vesicles beneath the plasma membrane with key roles for parasite motility and invasion (3). As a consequence of this, the phospholipid content of the infected RBC increases almost fivefold during intraerythrocytic development (4). Fatty acids, essential building blocks of membrane lipids, are largely taken up from the host, but due to the presence of a functional fatty acid synthesis type II system in the apicoplast, a non-photosynthetic plastid derived from algae, the parasite can also synthesize fatty acids *de novo*; this is particularly important for parasite development in the liver (5). Generation and homeostasis of membranes are based on a complex metabolic network, which not only requires *de novo* synthesis and acquisition but also phospholipid modification and recycling. Degradation of phospholipids is performed by phospholipases, which hydrolyze specific ester bonds in phospholipids and are classified into four groups, A, B, C, and D, based on their hydrolysis activity (6). Although phospholipases likely play key functions in *Plasmodium* cell biology, little is known about their role in the proliferation of the malaria parasite.

Here, we have performed a comprehensive functional screen of the phospholipase gene family during the intraerythrocytic asexual replication cycle of the most virulent malaria parasite species *Plasmodium falciparum*. Using conditional inactivation techniques, we further provide evidence for a physiological function of the phosphoinositide-specific phospholipase C (PI-PLC) during intracellular parasite maturation, long before its previously perceived role in parasite egress and invasion.

## RESULTS

### Gene deletion screening of the *Plasmodium* phospholipase family in asexual blood stages

We initiated the systematic functional analysis of *Plasmodium* phospholipases in *P. falciparum* asexual blood stages by first searching the *Plasmodium* genome for genes that encode for proteins containing putative lipase/phospholipase-related domains (Plasmodb.org [7]). This resulted in a list of 26 genes encoding enzymes with putative phospholipase function (Fig. S1) that also included the 22 putative phospholipases identified previously (6). For 19 of these 26 genes, there exists mass spectrometric evidence for expression in asexual blood-stage parasites (8 - 18). We, therefore, focused subsequent experiments on these 19 genes to obtain functional insights into their role in the erythrocytic parasite life cycle.

**Fig 1:**
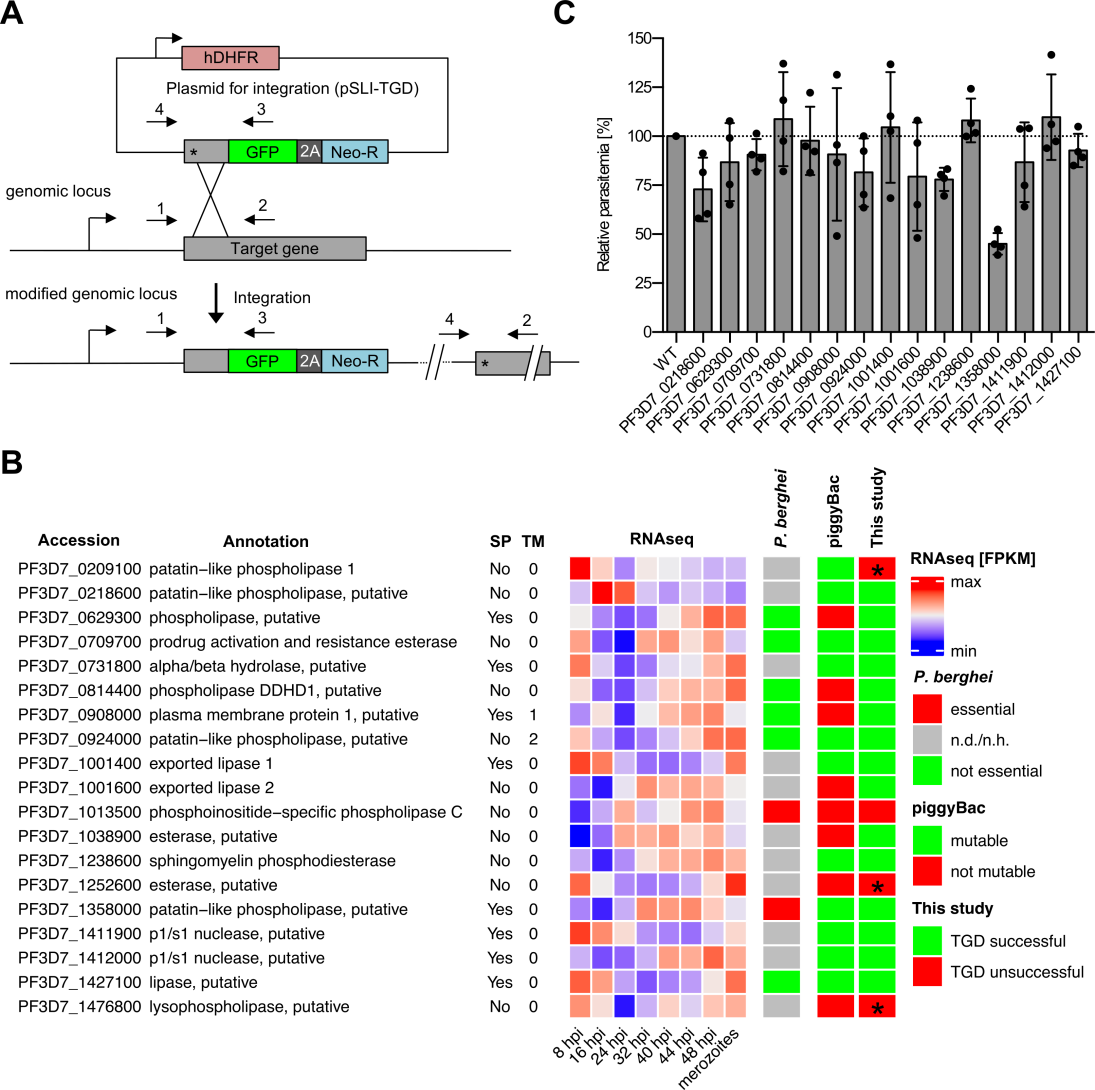
Gene disruption screen of the 19 predicted *P. falciparum* phospholipases expressed during blood-stage development. (**A**) Schematic of the SLI strategy used for TGD-based essentiality screening of the 19 putative phospholipases that show expression evidence in blood stages by mass spectrometry. Localization of primers used to detect successful integration of targeting constructs by PCR is indicated. Integration PCR results are displayed in Fig. S2. 2A, skip peptide; Neo-R, neomycin-resistance gene; asterisk, stop codon; arrows, promoters. (**B**) Results of the gene deletion screen compared to the results of genome-wide knockout (KO) screens in *P. falciparum* using piggyBac transposon-based mutagenesis (20) and in *P. berghei* (21), respectively. RNAseq expression data are derived from reference (22). For further details, see Fig. S1. SP, signal peptide; TM, transmembrane domain; n.d./n.h., not determined/no homolog in *P. berghei*. False-positive hits for which TGD was not successful but that were shown to be not essential for blood-stage development by other studies or by conditional KO approaches performed in this study are indicated with asterisks. (**C**) Flow cytometry-based growth analysis of synchronous phospholipase mutant parasite lines after two erythrocytic cycles in comparison to 3D7 WT parasites. The relative growth of each parasite line is shown in comparison to 3D7 WT parasites, the growth rate of which was normalized to 100% in each experiment. Shown are means ± SD of four independent growth experiments per parasite line. Raw parasitemia values are shown in Fig. S2.

We disrupted the expression of each gene by targeted gene disruption (TGD) using the selection-linked integration (SLI) system (19) (Fig. 1A). Of the 19 transfected targeting constructs, each designed to disrupt expression of the targeted gene, we obtained outgrowth of viable parasites displaying correct integration into their respective gene loci in 15 cases, indicating that the corresponding genes are not essential for *in vitro* parasite growth (Fig. 1B; Fig. S2A). Analysis of the obtained mutant lines for potential growth defects revealed that only the patatin-like phospholipase PF3D7_1358000 mutant consistently showed a reduction in the growth rate of ~50% in comparison to wild-type (WT) parasites over two parasite cycles, while all the other mutant parasite lines displayed no or very slightly reduced growth rates (Fig. 1C; Fig. S2B). For the remaining four putative phospholipase genes (PF3D7_0209100, PF3D7_1013500, PF3D7_1252600, and PF3D7_1476800), we consistently failed to obtain viable parasites harboring correctly integrated targeting plasmids, suggesting that these genes could be important or essential for the propagation of asexual blood-stage parasites (Fig. 1B). Collectively, this suggests a high level of functional redundancy within putative phospholipases during *in vitro* parasite replication within RBCs.

### PF3D7_1252600 and PF3D7_1476800 are not essential for parasite proliferation in RBCs

Our SLI-based gene disruption data suggest that PF3D7_1252600, PF3D7_1476800, PF3D7_0209100, and PF3D7_1013500 (PI-PLC) might be important for parasite growth within erythrocytes, given that we were not able to obtain viable parasites harboring correctly integrated targeting plasmids. For PF3D7_0209100, two previous studies revealed dispensability for asexual blood-stage parasite growth (23, 24); therefore, we analyzed the remaining three putative phospholipases in further detail.

PF3D7_1252600 is annotated as a putative esterase (PlasmoDB.org) and encodes a protein of 453 amino acids with a peak expression in merozoites and ring-stage parasites during asexual blood-stage development (22). It harbors a predicted α/β-hydrolase domain and lacks any transmembrane domains (Fig. S3A). In order to localize PF3D7_1252600 in the parasite, we appended a C-terminal spaghetti monster-Myc (smMyc) tag (25) using the SLI system and verified the genetic modification by PCR (Fig. S3B and C). Western blot analysis confirmed the expression of PF3D7_1252600-smMyc of the expected size in blood-stage parasites (Fig. S3D). Subsequent immunofluorescence analysis (IFA) revealed that PF3D7_1252600 mainly localizes to focal structures in the parasite that were not colocalizing with the micronemal marker AMA1 (Fig. S3E) or the rhoptry marker Rap1 (Fig. S3F). For functional characterization, we generated a conditional knockout (cKO) of PF3D7_1252600 based on the dimerizable Cre recombinase (DiCre) system (26, 27) and disrupted the endogenous gene within the functional α/β-hydrolase domain before the catalytic GXSXG motif (Fig. S3G and H). Gene KO, upon addition of rapalog (Rapa) to synchronous ring-stage parasites, was verified by excision PCR (Fig. S3I) and Western blot (Fig. S3J). However, conditional gene KO was not associated with any growth phenotype (Fig. S3K), suggesting that PF3D7_1252600 plays a redundant function in blood-stage proliferation.

We next focused our attention on PF3D7_1476800. This gene encodes a protein of 371 amino acids, containing a putative α/β-hydrolase domain, and shows peak expression in early schizonts during asexual blood-stage development (Fig. S4A) (22). PF3D7_1476800 was previously referred to as *P. falciparum* lysophospholipase 3 (*Pf*LPL3) and suggested to be important for the intraerythrocytic development of the parasite based on knockdown studies (28). To probe the physiological function of PF3D7_1476800, we appended a C-terminal triple hemagglutinin (HA) epitope and conditionally excised the majority of the α/β-hydrolase domain and the catalytic GXSXG motif using a DiCre-based cKO approach (Fig. S4B). The expected genetic modification was confirmed by PCR (Fig. S4C); however, our repeated attempts to confirm the expression of PF3D7_1476800 by IFA or Western blot failed. Rapamycin (RAP) treatment did result in efficient excision of PF3D7_1476800 based on PCR (Fig. S4D). Unexpectedly and in contrast to previous knockdown studies (28), this was not associated with any impairment of asexual blood-stage growth (Fig. S4E), indicating that PF3D7_1476800 plays a redundant function for blood-stage proliferation.

### PI-PLC has an essential role for parasite proliferation

**Fig 2:**
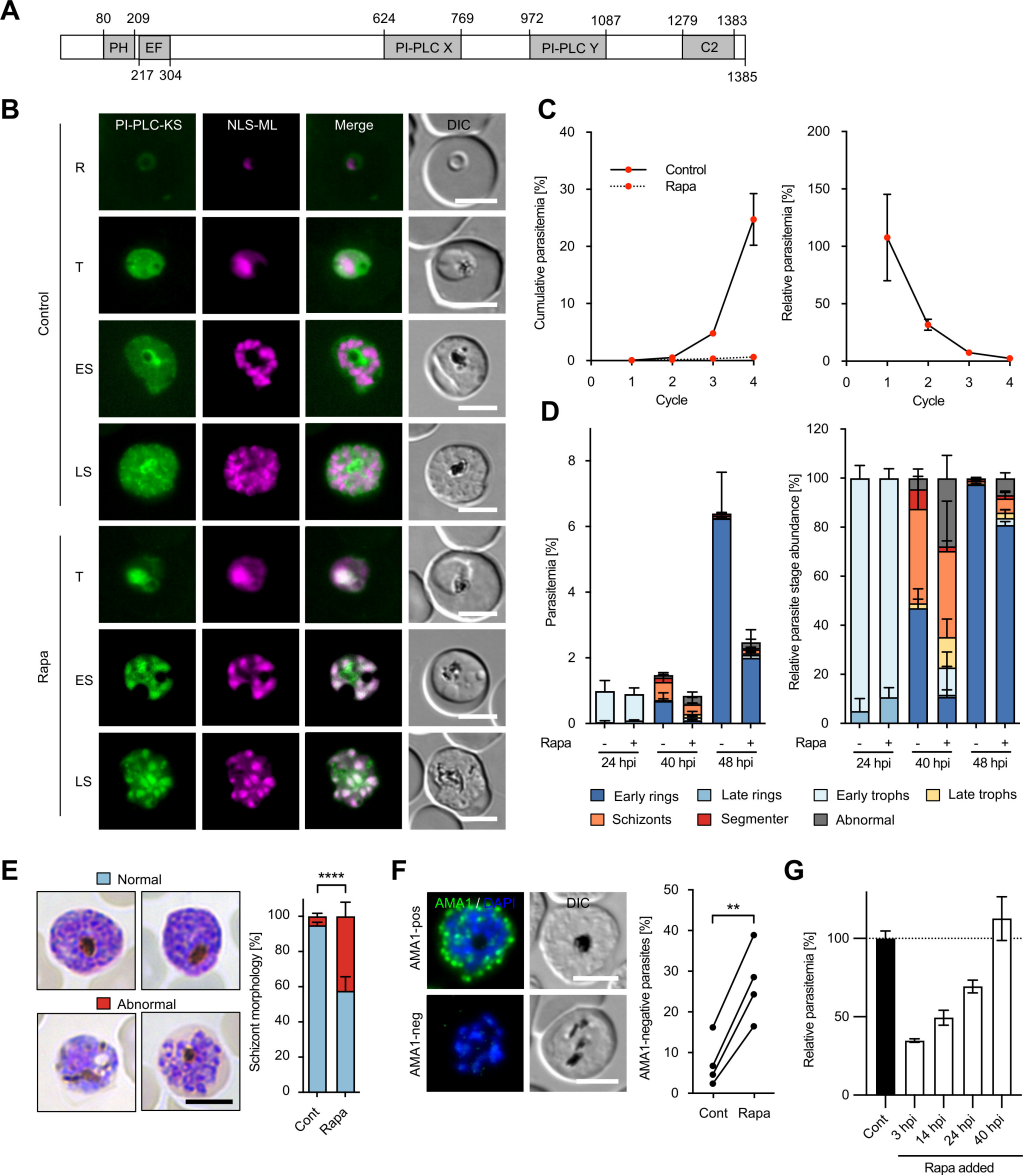
PI-PLC has an essential role in trophozoite and schizont development. (**A**) Schematic representation of the functional domains of PI-PLC. (**B**) Live cell microscopy of ring (R), trophozoite (T), early schizont (ES), and late schizont (LS) stages of PI-PLC-GFP-KS parasites expressing endogenously FKBP-GFP-tagged PI-PLC (green) in addition to a nuclear mislocalizer fused to mCherry (NLS-ML, magenta). Parasites were either untreated (control) or treated with Rapa to conditionally mislocalize the PI-PLC to the nucleus. DIC, differential interference contrast. Scale bars, 5 µm. (**C**) Growth over four erythrocytic cycles of PI-PLC-GFP-KS parasites treated with Rapa in comparison to untreated control parasites as determined by flow cytometry. For the calculation of relative parasitemia values (shown on the right), the parasitemia of Rapa-treated parasites was divided by the parasitemia of the respective untreated controls. Shown are means ± SD of three independent experiments. (**D**) Stage and parasitemia quantification of control and Rapa-treated PI-PLC-GFP-KS parasites at 24, 40, and 48 hpi. Shown are means ± SD of four independent experiments. (**E and F**) Schizont morphology and AMA1 expression of untreated and Rapa-treated PI-PLC-GFP-KS schizonts at 48 hpi, which were cultured in the presence of C2 for 8 hours to prevent egress. In panel **E**, schizont morphology was determined by microscopic examination of Giemsa-stained parasites. Shown are means ± SD of four independent experiments, in which a total of 156 control and 153 Rapa-treated schizonts were analyzed. Statistical evaluation was by unpaired Student’s *t*-test (****P < 0.0001). Representative images of normal and abnormal schizonts are shown on the left. In panel **F**, AMA1 expression was assessed by IFA. Shown are means ± SD of four independent experiments, in which a total of 306 control and 356 Rapa-treated schizonts were analyzed. Statistical analysis was by a paired Student’s *t*-test (**P < 0.01). Representative AMA1-positive and AMA1-negative schizonts are shown on the left. Scale bars, 5 µm. (**G**) Time course of PI-PLC inactivation. Synchronous PI-PLC-GFP-KS parasites were left untreated or treated with Rapa at various time points after infection. Parasitemia in the subsequent intraerythrocytic parasite cycle was determined by flow cytometry, and the parasitemia of untreated control parasites was set to 100%. Shown are means ± SD of four independent experiments.

Based on our SLI-based gene disruption data indicating that the single *P. falciparum* PI-PLC (PF3D7_1013500) might be critical for parasite growth (Fig. 1B), we next decided to further investigate the functional role of this putative enzyme. *P. falciparum* PI-PLC is 1,385 amino acids in length and contains all the functional domains typical for PI-PLC enzymes of the delta subclass, including (i) a lipid-binding pleckstrin homology-domain (residues 80–209); (ii) a calcium-binding EF-hand motif (residues 217–304); (iii) a catalytic domain consisting of an X (residues 624–769) and Y domain (residues 972–1,087); and (iv) a calcium/lipid-binding C2 domain (residues 1,279–1,383) (Fig. 2A) (29). To analyze the subcellular localization of PI-PLC and study its function, we made use of the conditional knocksideways system, which is based on the ligand-induced dimerization of the FK506-binding protein (FKBP) and the FKBP–rapamycin-binding (FRB) domain (19). For this, we first tagged the endogenous PI-PLC coding sequence by generating a C-terminal fusion to green fluorescent protein (GFP) flanked by two FKBP domains (Fig. S5). We then expressed in this parasite line a “mislocalizer” protein called NLS-ML, consisting of mCherry fused to an FRB domain and a nuclear localization signal (NLS). The addition of Rapa mediates heterodimerization of the NLS-ML and PI-PLC-GFP-FKBP proteins, removing the latter from its physiological site of action to the nucleus (19). The resulting parasite line, called PI-PLC-GFP-knocksideways (PI-PLC-GFP-KS), was used for subsequent localization and functional characterization.

Previous RNAseq studies have shown peak expression of the *pi-plc* gene during trophozoite and schizont development of the parasite (22). In accord with this, live microscopic examination of untreated PI-PLC-GFP-KS parasites revealed a GFP signal in trophozoite- and schizont-stage parasites that was mainly confined to the parasite cytoplasm. Interestingly, in mature schizonts, the signal appeared to partially surround developing daughter merozoites, suggesting a potential association of PI-PLC with the parasite plasma membrane (Fig. 2B, Control). Treatment of synchronous ring-stage PI-PLC-GFP-KS parasites with Rapa led to a rapid redistribution of the PI-PLC signal to the nucleus, as expected, leading to efficient colocalization with the NLS-ML signal (Fig. 2B, Rapa). To investigate the effects of this conditional PI-PLC mislocalization on parasite development, we compared the replication rates of untreated and Rapa-treated parasites over four erythrocytic cycles using flow cytometry. Consistent with the results of our SLI-based gene disruption screen (Fig. 1B), Rapa-treated parasites failed to replicate (Fig. 2C), confirming that PI-PLC is essential for blood-stage proliferation.

### PI-PLC is involved in parasite maturation

To determine the specific stage(s) in the erythrocytic developmental cycle affected by conditional mislocalization of PI-PLC, we monitored the development of tightly synchronized control and Rapa-treated PI-PLC-GFP-KS parasites by light microscopic examination of Giemsa-stained thin blood films. While parasite development appeared to be unaffected over the first 24 hours post RBC invasion (24 hpi), clear effects on parasite maturation were detected in Rapa-treated PI-PLC-GFP-KS parasites by 40 and 48 hpi (Fig. 2D). At 40 hpi, ~25% of Rapa-treated parasites were still at the trophozoite stage, in contrast to the untreated parasites at this time point, in which hardly any trophozoites were detectable. Furthermore, ~40% of the Rapa-treated parasites that formed schizonts displayed abnormal morphology. Together, these observations suggest that PI-PLC is involved in trophozoite and schizont development. Likely as a consequence of this, ring-stage parasitemia values at 40 and 48 hpi were reduced by more than 60% in the Rapa-treated parasites (Fig. 2D).

To analyze in further detail this potential function of PI-PLC during schizont development, we used an inhibitor of the parasite cGMP-dependent protein kinase G (PKG), called compound 2 (C2, which prevents egress), to synchronize parasites at the mature schizont stage (30). Examination of these C2-arrested schizonts revealed that more than 40% of Rapa-treated PI-PLC-GFP-KS parasites were dysmorphic (Fig. 2E). In line with this, analysis by IFA of the C2-arrested parasites showed that a high proportion of Rapa-treated parasites failed to express the late-stage specific marker AMA1 (Fig. 2F). To further investigate the function of PI-PLC over time, we also performed an experiment, in which we added Rapa not only to young rings (3 hpi) but also to later rings (14 hpi), trophozoites (24 hpi), and late schizonts (40 hpi), followed by measuring the parasitemia in the subsequent intraerythrocytic parasite cycle. Interestingly, this revealed that only Rapa treatment of PI-PLC-GFP-KS parasites during ring or trophozoite development impaired parasite replication, while no inhibition of parasite growth was observed by Rapa treatment of schizonts (Fig. 2G). This further supports an additional key function of PI-PLC before egress and invasion and highlights the crucial role of PI-PLC in intraerythrocytic parasite maturation.

### Conditional disruption of PI-PLC confirms its essentiality for *P. falciparum* schizont development

**Fig 3:**
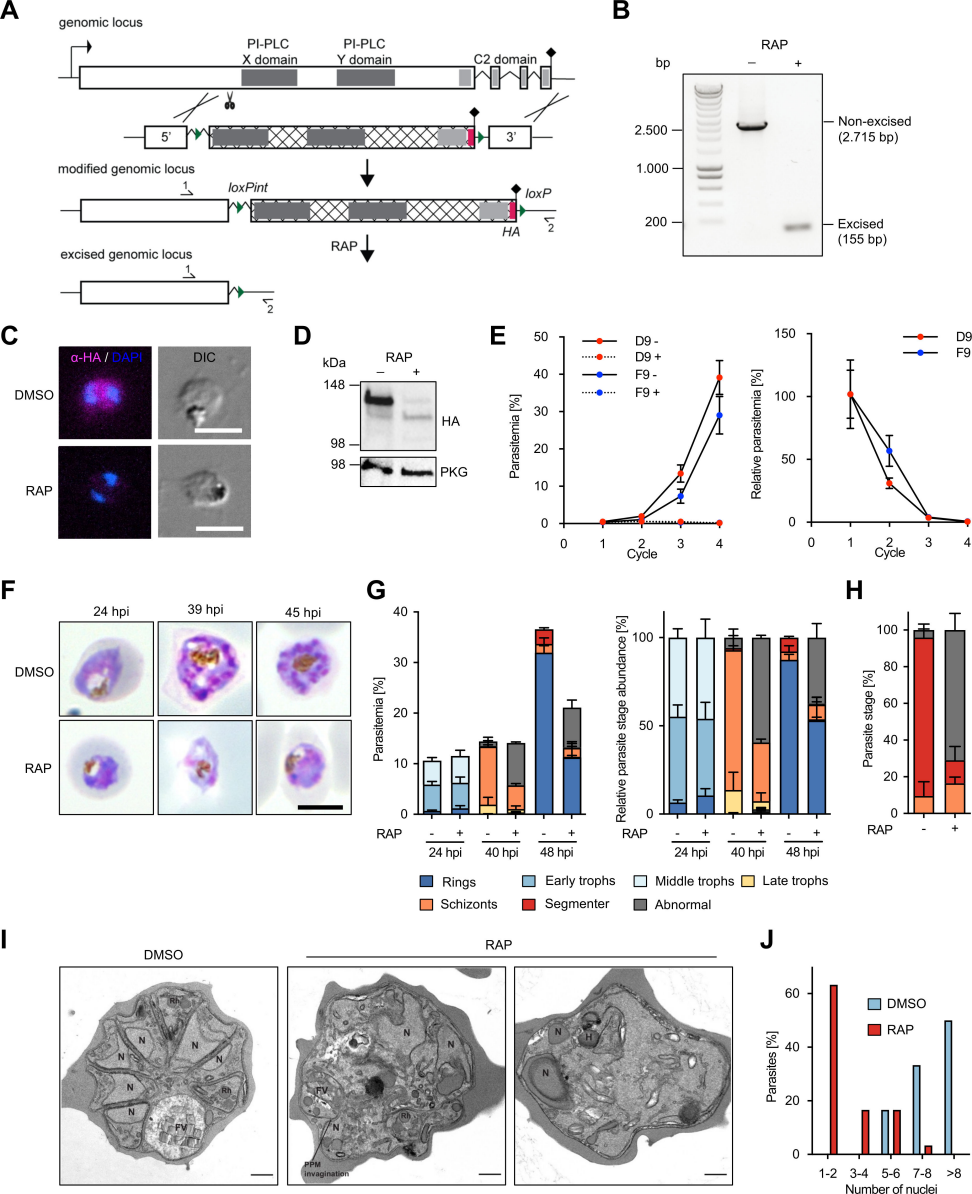
Conditional gene disruption confirms essentiality of PI-PLC. (**A**) Schematic of the strategy used to generate a conditional PI-PLC KO line (PI-PLC:HA:loxPint). The X and Y catalytic domains (dark gray) and the lipid-binding C2 domain (light gray) were floxed by introducing an upstream *loxP*-containing intron (loxPint) and a second *loxP* site downstream of the translational stop site (lollipop). Site of targeted Cas9-mediated double-stranded DNA break (scissors), left and right homology arms for homology-directed repair (5′ and 3′), introduced *loxP* sites (arrow heads), recodonized sequences (hatched), and 3xHA epitope (red) are indicated. RAP-induced DiCre-mediated excision results in the removal of the functional domains. Primers 1 and 2 (half arrows) were used for diagnostic PCR to assess excision. (**B**) Confirmation of efficient gene excision by PCR. Samples were taken at 12 hours post RAP or mock (dimethyl sulfoxide [DMSO]) treatment of ring-stage parasites. Expected PCR amplicon sizes from non-excised and excised parasites are shown. Shown is one representative experiment (of five independent experiments). (**C**) IFA images of DMSO- or RAP-treated PI-PLC:HA:loxPint trophozoite-stage parasites. Parasites were fixed at 33 hpi and stained with an anti-HA-antibody (magenta). DAPI-stained nuclei are shown in blue. DIC, differential interference contrast. Scale bars, 5 µm. (**D**) Western blot of mature schizonts (45 hpi) showing successful RAP-induced ablation of PI-PLC-3xHA expression in the erythrocytic cycle of RAP addition. PKG served as a loading control. Note that PI-PLC-3xHA in DMSO-treated parasites runs slightly lower as compared to its calculated molecular weight of 167,4 kDa. (**E**) Replication of DMSO-treated (solid line) and RAP-treated (dashed line) parasites from two clonal lines of PI-PLC:HA:loxPint over four erythrocytic cycles. For the calculation of relative parasitemia values (shown on the right), the parasitemia of RAP-treated parasites was divided by the parasitemia of respective DMSO-treated control parasites. Shown are means ± SD of three biological replicates (different blood sources). (**F**) Giemsa-stained images of DMSO- and RAP-treated PI-PLC:HA:loxPint parasites at 24, 39, and 45 hpi. Representative images of two independent experiments are shown. Scale bars, 5 µm. (**G**) Stage and parasitemia quantification of DMSO- and RAP-treated PI-PLC:HA:loxPint parasites at 24, 40, and 48 hpi. Shown are means ± SD of three biological replicates. (**H–J**) Morphological analysis of DMSO- or RAP-treated PLC:HA:loxPint parasites that were allowed to mature to egress-stalled schizonts from 46 to 49 hpi in the presence of C2. In panel **H**, parasite morphology was assessed on Giemsa-stained blood smears. Shown are means ± SD of three independent experiments. Color code same as in panel **G**. In panels **I** and **J**, parasite morphology was assessed using transmission electron microscopy. Representative images of DMSO- and RAP-treated parasites are displayed in panel **I**, and a quantification of nuclei is shown in panel **J**. Results are representative of 18 DMSO- and 30 RAP-treated analyzed parasites. N, nucleus; FV, food vacuole; Rh, rhoptries; H, hemoglobin-containing cytostome. Scale bar, 500 nm.

The knocksideways system is a powerful tool to study the function of essential proteins that do not enter the secretory pathway (19). However, under conditions where mislocalization is not 100% efficient, varying amounts of target protein can remain at the site of action and, therefore, functional. We, thus, decided to further probe the function of PI-PLC using a DiCre-based cKO approach (26, 27). For this, a 3′-proximal segment of the *pi-plc* open reading frame encoding the predicted catalytic core of PI-PLC (the predicted X and Y domains), as well as the calcium/lipid-binding C2 domain, was targeted for excision by replacing the endogenous gene segment with a synthetic modified version using Cas9-enhanced homologous recombination. The modified sequence incorporated (i) a short synthetic intron containing a *loxP* site (loxPint) upstream of the catalytic domains; (ii) the recodonized version of the segment encoding the WT amino acid sequence but with altered codon usage; (iii) a C-terminal triple-HA epitope tag just preceding the translational stop codon; and (iv) a second *loxP* site immediately following the translational stop codon (Fig. 3A). The genetic modification was performed in the B11 *P. falciparum* line (31), which stably expresses DiCre recombinase. DiCre-mediated excision of the floxed sequence was expected to result in conditional inactivation of PI-PLC due to the deletion of its catalytic domains. The transgenic parasites (called PI-PLC:HA:loxPint) were cloned by limited dilution, and two clonal parasite lines (D9 and F9) were isolated. The expected genetic modifications in both clones were confirmed by diagnostic PCR (Fig. S6). RAP treatment of tightly synchronized ring-stage PI-PLC:HA:loxPint parasites resulted in the anticipated truncation of PI-PLC within the same erythrocytic cycle, as detected by PCR (Fig. 3B), IFA (Fig. 3C), and Western blot (Fig. 3D). To initially assess the viability of the resulting PI-PLC-null mutants, growth of RAP- and mock-treated cultures of the two PI-PLC:HA:loxPint clonal lines was monitored over the course of four erythrocytic cycles. PI-PLC-null parasites failed to proliferate, confirming our knocksideways-based results that PI-PLC is crucial for viability during asexual blood-stage replication of *P. falciparum* (Fig. 3E). For more in-depth characterization, the F9 PI-PLC:HA:loxPint clone was used in all subsequent experiments.

Intracellular development of PI-PLC-null mutants within the erythrocytic cycle of RAP treatment was studied by microscopic examination of Giemsa-stained parasites. Similar to our results obtained with the knocksideways system, this revealed that PI-PLC-null mutants underwent apparently normal growth until late trophozoite/early schizont stage, after which they developed morphological abnormalities during schizont development (Fig. 3F through H). To further analyze this, we performed transmission electron microscopy of C2-arrested parasites. This revealed that more than 60% of PI-PLC-null parasites possessed poorly defined subcellular organelles, and only one to two nuclei were visible in the sections (rather than the seven or more nuclei which were observed in about 80% of mock-treated control schizonts) (Fig. 3I and J). Despite these developmental defects, hemoglobin-containing cytostomes and hemozoin crystals were evident in the digestive vacuole of the PI-PLC-null parasites, suggesting that the mutants retained the capacity to internalize and digest hemoglobin. Around 17% of PI-PLC-null parasites showed the formation of three to four nuclei, well-formed rhoptries, and parasite plasma membrane invaginations, pointing to the start of merozoite formation. However, we were unable to find more than a few well-segmented schizonts in the PI-PLC-null samples, in contrast to the majority of the mock-treated parasites, which formed well-segmented schizonts with clearly defined merozoites (Fig. 3I and J). Taken together, we concluded that lack of PI-PLC caused a severe phenotype during schizont development, suggesting that PI-PLC-mediated activity is critical for intraerythrocytic parasite maturation.

### Phosphoinositide analysis of PI-PLC-deficient parasites

**Fig 4:**
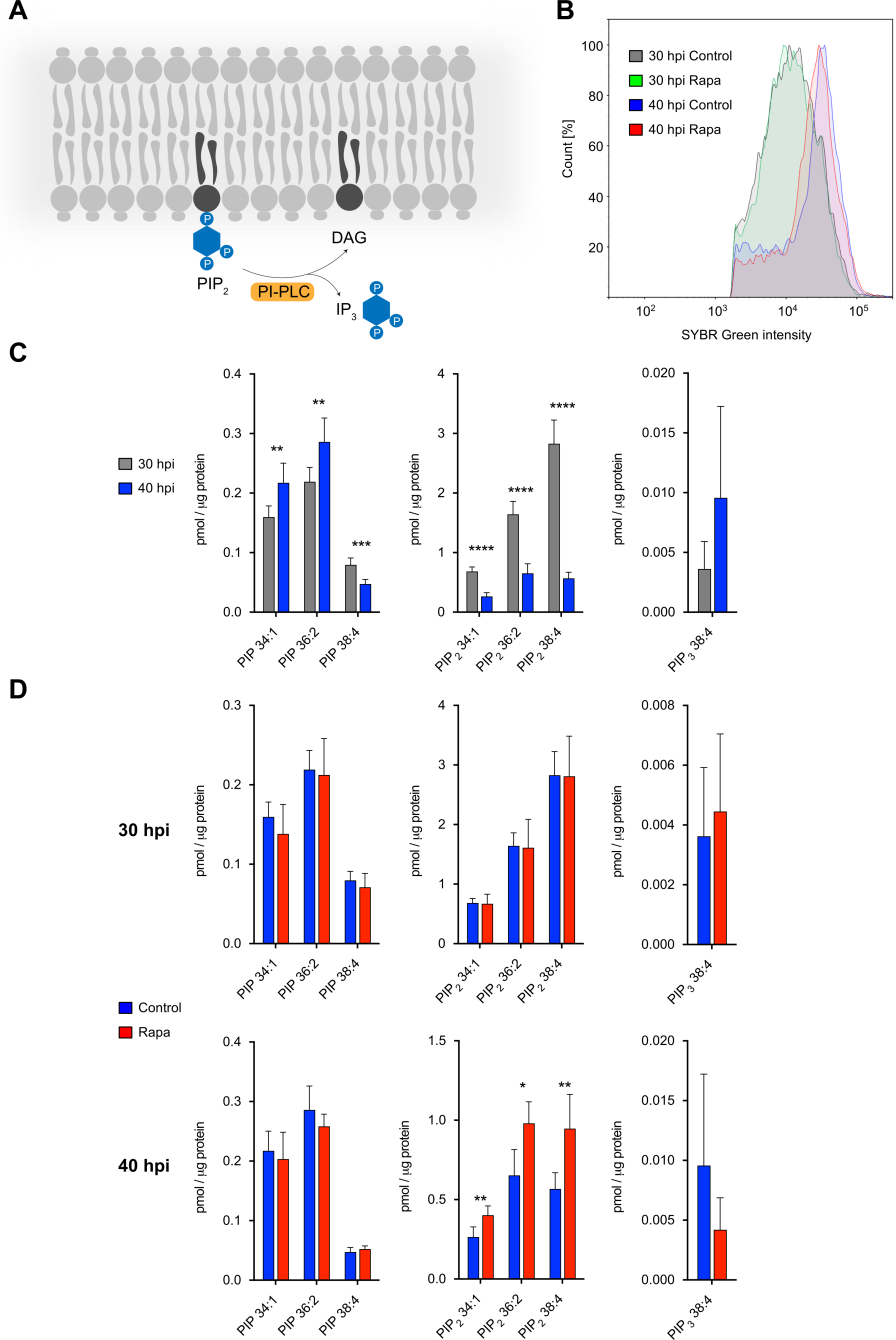
Conditional inactivation of PI-PLC impairs phosphoinositide homeostasis. (**A**) Schematic of PI-PLC-mediated cleavage of PIP_2_ to IP_3_ and DAG. (**B**) Fluorescence intensity of SYBR Green-stained control and Rapa-treated parasites at 30 and 40 hpi, respectively, as determined by flow cytometry. Shown is one representative out of eight biological replicates. (**C and D**) Lipidomic analysis of phosphoinositides in untreated and Rapa-treated PI-PLC-GFP-KS parasites. Synchronous parasites were grown in the absence (control) or presence of Rapa and harvested at 30 and 40 hpi. After releasing parasites from their host cell using saponin treatment, lipids were isolated and subjected to lipidomic analysis. The abundance of phosphoinositides was quantified and normalized to the amount of protein that was determined in parallel. Phosphoinositide abundance in untreated control parasites at 30 and 40 hpi is shown in panel **C**, while phosphoinositide abundance at 30 and 40 hpi in control and Rapa-treated parasites is displayed in panel **D**. Data are based on eight biological replicates per time point and treatment. Means ± SD are shown. Statistical significance was assessed with paired Student’s *t*-test. All statistically significant differences are indicated (*P < 0.05, **P < 0.01, ***P < 0.001). For source data of this figure, please see Supplemental file 2.

Given that PI-PLC is expected to cleave phosphatidylinositol bisphosphate (PIP_2_) to inositol trisphosphate (IP_3_) and diacylglycerol (DAG) (Fig. 4A) (32), we next tested whether the maturation phenotype upon conditional inactivation of PI-PLC is associated with a perturbation of lipid homeostasis. To this aim, we subjected untreated and Rapa-treated PI-PLC-GFP-KS trophozoites (30 hpi) and schizonts (40 hpi) to lipidomic analysis, after confirming by flow cytometry that parasite stages between the analyzed untreated and Rapa-treated parasites at both time points were relatively comparable, apart from a slightly reduced DNA content of Rapa-treated schizonts (Fig. 4B). In addition to a general lipidomic analysis of PI-PLC-deficient parasites covering 237 lipid species (Supplemental file 2) and the major abundant lipid classes (Fig. S7), we focused our attention on phosphoinositides, which have not been studied in detail in malaria parasites yet, as these were the expected primary substrates of PI-PLC. Interestingly, when we compared phosphoinositide levels between untreated parasites at 30 and 40 hpi, we saw a substantial downregulation of all quantified PIP_2_ species in schizont stages, while more moderate changes were observed for phosphatidylinositol monophosphate (PIP) species (Fig. 4C). Comparing untreated and Rapa-treated PI-PLC-GFP-KS parasites at both time points revealed no significant difference in phosphoinositide levels in trophozoites (Fig. 4D, upper panels). Remarkably, there was a significant upregulation of all measured PIP_2_ species in Rapa-treated schizont stages, while no significant differences were observed for phosphatidylinositol monophosphate species and phosphatidylinositol trisphosphate (PIP_3_) (Fig. 4D, lower panels). Collectively, these data support the conclusion that PI-PLC, indeed, mediates cleavage of PIP_2_ in schizont-stage parasites, although we currently cannot fully exclude that the observed differences in PIP_2_ might, to a certain extent, also be caused by secondary effects related to the differences in parasite development and morphology that inevitably occur upon PI-PLC inactivation.

## DISCUSSION

Phospholipases are ubiquitously occurring enzymes that catalyze the cleavage of phospholipid molecules. As a result, these enzymes are involved in diverse physiological processes, including the remodeling of cellular membranes, lipid-mediated signal transduction processes, cell proliferation, and virulence (6).

The focus of this study was a systematic functional characterization of the 19 putative *P. falciparum* phospholipase genes for which mass spectrometry data indicated expression during asexual blood-stage development. We first used an SLI-based gene disruption strategy to show that out of the 19 genes, 15 could be readily disrupted without loss of parasite viability, pointing to a high level of redundancy within putative phospholipases of *P. falciparum*. When we analyzed the proliferation of these 15 mutants, only parasites lacking the patatin-like phospholipase (PNPLA) PF3D7_1358000 showed a growth defect of about 50% over two intraerythrocytic cycles. PNPLAs are highly conserved enzymes of prokaryotic and eukaryotic organisms with a broad physiological role (33). Apart from PF3D7_1358000, the *P. falciparum* genome encodes three additional putative PNPLA enzymes (PF3D7_0209100, PF3D7_0218600, and PF3D7_0924000), all of which appear to be non-essential for asexual blood-stage multiplication [Fig. 1B; (23, 24)]. Interestingly, PNPLA1 (PF3D7_0209100) has been shown to be critical in gametocyte induction (23) and gametogenesis (24).

For 4 of the 19 phospholipases analyzed in our targeted gene disruption screen, no transgenic KO parasites were obtained, suggesting a possible critical role in parasite growth. However, targeting two of these, PF3D7_1252600 and PF3D7_1476800, using a DiCre-based cKO approach did not reveal any defect in parasite proliferation, indicating that they are not essential for parasite replication. This was, in particular, unexpected for PF3D7_1476800, given that a previous study localized the protein to the parasitophorous vacuole and showed that its conditional knockdown using the glmS ribozyme system disrupts parasite development from trophozoites to schizonts, leading to a strong decrease in merozoite progeny (28). The reasons for these interesting phenotypic differences remain to be discovered but could be connected to the differences in conditional reverse genetics tools used in these two studies (glucosamine-induced mRNA degradation vs. RAP-induced gene excision). For the third candidate, PNPLA1, two previous studies similarly revealed dispensability for asexual parasite growth (23, 24). Together, this indicates that these three candidates are false-positive hits of our SLI-based gene disruption screen, further underlining the high level of redundancy among putative *Plasmodium* phospholipases. Whether some of this functional redundancy is due to compensatory mechanisms that are induced upon gene disruption (e.g., overexpression of functionally equivalent other phospholipases) remains to be elucidated. Nevertheless, functional redundancy among phospholipases in pathogens is well established, including an excellent example in *Listeria monocytogenes*, where individual disruption of two phospholipases resulted in moderate effects on infectivity to mice (2- to 20-fold reduction), whilst simultaneous disruption of both phospholipases in combination led to a severely impaired infectivity (500-fold reduction) (34). Generating double or even triple KO mutants in the future may reveal the functional interplay between putative phospholipases in the malaria parasite, and the collection of parasite lines generated in this study will be a useful resource for this purpose. One potential starting point for these approaches could be the KO parasites of the PNPLA PF3D7_1358000, given their slow proliferation rate phenotype.

The combined essentiality data that we obtained in this study for putative *Plasmodium* phospholipases are overall in agreement with a genome-wide saturation mutagenesis screen in *P. falciparum* (20) (Fig. 1B). An exception were the seven putative phospholipases, which we identified to be non-essential for blood stage proliferation by application of targeted gene disruption or DiCre-based cKO approaches, although they were predicted to be essential by the genome-wide KO screen (20). This underlines the need to verify global-scale screening data at the single gene level.

Some of the non-essential putative phospholipases identified in our work have been previously studied. The homolog of PF3D7_0629300 in the rodent malaria model *P. berghei* (PBANKA_1128100) exhibits phospholipase and membranolytic activity *in vitro* and has been implicated in cell traversal by sporozoites and disruption of the liver-stage PVM during parasite egress from hepatocytes (35, 36). The sphingomyelin phosphodiesterase (PF3D7_1238600) was identified as a PLC able to hydrolyze sphingomyelin and lysocholinephospholipids, and inhibitor studies using scyphostatin were used to argue for an essential role of the enzyme during asexual growth (37). Our study now provides reverse genetic evidence that PF3D7_1238600 is dispensable for parasite proliferation, suggesting that scyphostatin has additional targets within the parasite. PF3D7_0709700, previously designated as prodrug activation and resistance esterase *Pf*PARE, was shown to have esterase activity to activate esterified pepstatin, a peptidyl inhibitor of malarial aspartyl proteases (38). *Pf*PARE active site mutants were not impaired in asexual blood-stage growth (38), mirroring our successful gene disruption. Finally, the two non-essential putative lysophospholipases, PF3D7_1001400 and PF3D7_1001600, contain a PEXEL motif and were shown to be exported into the host RBC, although their molecular functions have not yet been determined (39).

The last phospholipase candidate in our targeted gene disruption screen, for which we consistently failed to obtain viable parasites harboring correctly integrated targeting plasmid, was the PI-PLC of the parasite. PI-PLCs are phosphodiesterases that participate in PIP_2_ metabolism and lipid signaling pathways in a Ca^2+^-dependent manner (32). Several previous studies have suggested that PI-PLC is essential for parasite blood-stage proliferation, but definitive genetic evidence for this has been lacking. Earlier work has shown that the PI-PLC homolog in *P. berghei* (PBANKA_1211900) is refractory to genetic deletion (29). Studies have suggested that PI-PLC activity is involved in multiple processes ranging from gametocyte development and sporozoite motility to egress and invasion of merozoites by regulating Ca^2+^ release (40 - 43). PI-PLC was shown to likely act downstream of the parasite protein kinase G, which regulates egress and activity of which promoted hydrolysis of the PI-PLC substrate PIP_2_ (44). However, all of these studies relied on the use of the small compound inhibitor U73122, for which the degree of selectivity for PI-PLC is unclear, given that in numerous other systems the compound has the potential to modulate Ca^2+^ homeostasis independently of PI-PLC inhibition (45 - 47).

Using two distinct conditional gene-targeting approaches, we now provide genetic evidence that PI-PLC is essential for *P. falciparum* asexual blood-stage proliferation. Both conditional inactivation techniques resulted in a defect in the development of schizonts. The maturation phenotype of our PI-PLC-deficient parasites is reminiscent of that seen in the related apicomplexan parasite *Toxoplasma gondii*, where conditional ablation of *Tg*PI-PLC caused significant morphological abnormalities during lytic stage growth (48). Together, these findings, therefore, support a similar function for PI-PLC in daughter cell formation in these two apicomplexan genera. This occurs long before the previously proposed role of PI-PLC in egress and invasion.

To probe the physiological role of PI-PLC in lipid homeostasis, we performed lipidomic analysis of trophozoites and schizonts, in which we conditionally inactivated PI-PLC. We concentrated on phosphoinositides, which are, in general, very difficult to analyze by lipidomics approaches due to their low abundance and the fact that they yield only low levels of detectable ions in the mass spectrometry process (49). By applying a recently developed method for the quantification of phosphoinositides that is based on TMS-diazomethane to induce methylation of phosphate groups in phosphoinositides (50, 51), we were able to overcome these limitations and could provide, for the first time, a detailed lipidomics-based analysis of phosphoinositide species in malaria parasites. Interestingly, this revealed higher PIP_2_ levels in schizonts missing functional PI-PLC in comparison to unmodified parasites, supporting PI-PLC-mediated cleavage of PIP_2_ in schizonts. Apart from their roles in signaling processes, phosphoinositides, including PIP_2_, play key functions for membrane organization by regulating vesicular trafficking or by controlling the non-vesicular exchange of lipids between membranes, for example (52). It is, thus, reasonable to discuss that the potentially dysregulated PIP_2_ levels that we detected upon PI-PLC depletion may have a major influence on membrane homeostasis, which may also explain some of the other defects we observed upon PI-PLC inactivation such as the formation of schizonts with abnormal morphology. In line with this, conditional inactivation of PI-PLC was associated with the dysregulation of several structural membrane lipids in schizonts (Supplemental file 2; Fig. S7), although it is possible that these lipidome alterations might also be caused by secondary effects related to differences in parasite maturation due to functional inactivation of PI-PLC.

In conclusion, our study provides a systematic functional analysis of phospholipases in the clinically relevant blood stages of *P. falciparum*. In addition, it identifies PI-PLC as an essential regulator of parasite maturation and thereby highlights parasite phosphoinositide metabolism as a potential target of novel therapeutic approaches.

## MATERIALS AND METHODS

### Cloning of SLI-based constructs

For generation of SLI-based TGD constructs, 312–954 bp immediately downstream of the start ATG of the target genes were amplified by PCR, starting with a stop codon, to serve as homology regions for single-crossover-based integration. PCR products were cloned using NotI/MluI into pSLI-TGD (19) to generate the final targeting plasmids.

For generation of the endogenous tagging construct pSLI-PF3D7_1252600-smMyc, the smMyc sequence was amplified from addgene plasmid #59757 (pCAG-smFP Myc, gift from Loren Looger) (25) using primer smMyc-fw/smMyc-rev and cloned into pSLI-TGD (19) using MluI/SalI, thereby replacing the GFP coding sequence with smMyc. Into the resulting plasmid pSLI-smMyc, we introduced via NotI/AvrII the C-terminal 816 bp of the PF3D7_1252600 coding sequence (starting with a stop codon), previously amplified by PCR using primers PF3D7_1252600-tag-fw/PF3D7_1252600-tag-rev.

For generation of PF3D7_1252600-cKO parasites, a plasmid was synthesized (GeneScript, Piscataway, NJ, USA) containing (i) a 324-bp targeting sequence corresponding to the first 324 bp after the start ATG of the PF3D7_1252600 coding sequence followed by a 2xMyc tag; (ii) a loxP site within an artificial intron (loxPint) followed by a T2A skip peptide; and (iii) the recodonized full-length PF3D7_1252600 coding sequence. To generate the final targeting plasmid pSLI-PF3D7_1252600-loxP, the aforementioned sequence was cloned via NotI/KpnI into pSLI-PfPMRT1-loxP (53).

For generation of the PI-PLC knocksideways construct pSLI-PF3D7_1013500-KS, the C-terminal 985 bp of the *pi-plc* gene were amplified by PCR using primers PF3D7_1013500-tag-fw/PF3D7_1013500-tag-rev, starting with a stop codon, and cloned into pSLI-sandwich (19) using NotI/AvrII.

Phusion high-fidelity DNA polymerase (New England BioLabs, Ipswich, MA, USA) was used for all plasmid constructions, and all plasmid sequences were confirmed by Sanger sequencing. For sequences of oligonucleotides and other synthetic DNA used in this study, see Supplemental file 1.

### Cloning of plasmids for cKO of PF3D7_1476800 and PI-PLC

Gene segments containing the catalytic domains of PF3D7_1476800 and the *pi-plc* gene were replaced by a synthetic, modified version using Cas9-enhanced homologous recombination by transfecting a guide plasmid and a linearized repair plasmid into the DiCre-expressing *P. falciparum* line B11 (31). A 1,040- and 2,417-bp long gene segment were chosen in PF3D7_1476800 and *pi-plc,* respectively.

Two single-guide RNA (sgRNA) inserts per target gene were generated by annealing oligo pairs PF3D7_1476800_gRNA01.F/PF3D7_1476800_gRNA01.R and PF3D7_1476800_gRNA02.F/ PF3D7_1476800_gRNA02.R for PF3D7_1476800, and PF3D7_1013500_gRNA01.F/ PF3D7_1013500_gRNA01.R and PF3D7_1013500_gRNA02.F/ PF3D7_1013500_gRNA02.R for PI-PLC, which were subsequently ligated into the BbsI-digested plasmid pDC2-Cas9-hDHFRyFCU plasmid (54) which contains sequences encoding Cas9, sgRNA, and the drug selectable marker hDHFR (human dihydrofolate reductase)/yFCU (yeast cytosine deaminase/uridyl phosphoribosyl transferase).

Repair plasmids were designed such that they had (i) ~500 bp native sequences on either side of the targeted gene segment to serve as homology arms; (ii) a short synthetic intron containing a *loxP* site (loxPint) upstream of the targeted gene segment; (iii) the recodonized version of the targeted gene segment with the PAM sites destroyed; (iv) a 3xHA epitope tag just prior to the gene translational stop codon; and (v) another *loxP* site following the translational stop codon.

To target PI-PLC, the above-designed construct was synthesized as two parts (2,866 and 791 bp) and combined by restriction-ligation (using HindIII and XhoI enzymes) to create pREP-piplc-3HA-loxPint.

Synthetic gene constructs were synthesized by GeneArt (Thermo Fisher Scientific, Waltham, MA, USA). Phusion high-fidelity DNA polymerase (New England BioLabs, Ipswich, MA, USA) was used for all plasmid constructions, and all plasmid sequences were confirmed by Sanger sequencing. For sequences of all primers and synthetic gene constructs, see Supplemental file 1.

### *P. falciparum* culture

Blood stages of 3D7 *P. falciparum* parasites and transgenic derivates were cultured in human RBCs, which were obtained from commercially purchased anonymous blood concentrates from the blood bank of the University Medical Center Hamburg-Eppendorf (Approval number 10569a/96-1). Cultures were maintained at 37°C in an atmosphere of 90% nitrogen, 5% carbon dioxide, and 5% oxygen (DiCre-based PF3D7_1476800 and PI-PLC cKO lines) or in an atmosphere of 94% nitrogen, 5% carbon dioxide, and 1% oxygen (all other parasite lines) using RPMI complete medium containing 0.5% Albumax according to standard procedures (55). For growth and lipidomic analysis of PI-PLC-GFP-KS parasites, medium additionally contained 2 mM choline. All work was performed according to the appropriate legal and work place safety regulations.

### Generation of SLI-based parasite lines

For transfection of constructs, Percoll (GE Healthcare, Chicago, IL, USA)-enriched synchronized mature schizonts of 3D7 parasites were electroporated with 50 µg of plasmid DNA using a Lonza Nucleofector II device (56). Transfectants were selected in medium supplemented with 3 nM WR99210 (Jacobus Pharmaceuticals, Princeton, NJ, USA), 0.9 µM DSM1 (BEI Resources, NIAID, NIH), or 2 µg/mL blasticidin S (Thermo Fisher Scientific, Waltham, MA, USA). For generation of stable integrant cell lines, parasites containing the episomal plasmids selected with WR99210 were grown with 400 µg/mL Neomycin/G418 (Sigma, St. Louis, MO, USA) to select for transgenic parasites carrying the desired genomic modification as described previously (19). Each WR-resistant parasite culture was routinely placed under neomycin selection in three independent experiments using three culture dishes each time and was followed up for 60 days to monitor the appearance of viable transgenic parasites (expected to represent parasites in which the targeted gene was disrupted). Successful integration was confirmed by diagnostic PCR using FIREpol DNA polymerase (Solis BioDyne, Tartu, Estonia). For primer sequences, see Supplemental file 1. For generation of PF3D7_1252600-cKO parasites, transgenic parasites were transfected with pSkipFlox (19) to episomally express the DiCre recombinase.

### Generation of PF3D7_1476800 and PI-PLC cKO parasites

PF3D7_1476800 and PI-PLC cKO parasites are based on the DiCre-expressing *P. falciparum* clone B11, derived from the 3D7 parasite line (31). Two transfections (one per guide RNA) were performed. Mature schizonts enriched using Percoll (GE Healthcare, Chicago, IL, USA) were electroporated with 20 µg of guide plasmid and 60 µg of linearized repair plasmid using an Amaxa 4D electroporator and P3 Primary cell 4D Nucleofector X Kit L (Lonza, Basel, Switzerland) using program FP158 as described (26). Twenty-four hours post transfection, the culture medium was replaced with fresh medium containing WR99210 (2.5 nM), which was withdrawn after 4 days. Once drug-resistant parasites appeared (in about 2 weeks), they were cloned by limiting dilution using a plaque-based method (57). Successful integration was confirmed by diagnostic PCR using GOtaq Hot Start Green Master Mix (Promega, Fitchburg, WI, USA). For primer sequences, see Supplemental file 1.

### Fluorescence microscopy

For staining of nuclei, parasites were incubated with 1 µg/mL DAPI (Sigma, St. Louis, MO, USA) in culture medium for 15 minutes at 37°C. PI-PLC cKO parasites were imaged using a Nikon Eclipse Ni-E widefield microscope equipped with a Hamamatsu C11440 digital camera and a 100×/1.45 NA oil immersion objective. All other parasite lines were imaged on a Leica D6B fluorescence microscope equipped with a Leica DFC9000 GT camera and a Leica Plan Apochromat 100×/1.4 oil objective. Image processing was performed using ImageJ.

### Western blot

For Western blot analysis of PF3D7_1252600-smMyc and PF3D7_1252600-cKO parasites, protein samples were resolved by SDS-PAGE and transferred to nitrocellulose membranes (LICOR). Membranes were blocked in 5% milk in TBS-T followed by incubation in the following primary antibodies that were diluted in TBS-T containing 5% milk: rabbit anti-Myc (Cell Signalling Technology, Danvers, MA, USA, #2272, 1:1,000), rat anti-HA mAb 3F10 (Sigma, St. Louis, MO, USA, 1:1,000), and mouse anti-GAPDH (58) (1:2,000). After washing three times in TBST-T, membranes were incubated in similarly diluted secondary antibodies: goat anti-rabbit-800CW (LICOR, Lincoln, NE, USA, 1:10,000), goat anti-mouse-680RD (LICOR, Lincoln, NE, USA, 1:10,000), and goat anti-rat-800CW (LICOR, Lincoln, NE, USA, 1:10,000). Subsequently, membranes were washed another three times with TBST-T and scanned on a LICOR Odyssey FC imager.

For Western blot analysis of PI-PLC-cKO parasites, parasites were Percoll enriched, washed, and lysed with saponin. The resulting parasite pellets were solubilized in five volumes of a denaturing solubilization buffer (1% [wt/wt] SDS in 50 mM Tris-HCl, pH 8.0, 5 mM EDTA, 1 mM PMSF] with sonication. Samples were immediately boiled for 5 minutes, clarified by centrifugation at 12,000 × *g* for 20 minutes, and subjected to SDS-PAGE. Proteins were transferred to nitrocellulose membranes. Membranes were then blocked in 3% BSA in PBS containing 0.2% Tween 20 before staining with rat anti-HA mAb 3F10 (Sigma, St. Louis, MO, USA, diluted 1:1,000) primary antibody in blocking buffer and then incubated with biotin-conjugated anti-rat antibody (Roche, Basel, Switzerland, diluted 1:8,000) in blocking buffer followed by horseradish peroxidase-conjugated streptavidin (Sigma, St. Louis, MO, USA, diluted 1:10,000). Antibody binding was detected using an Immobilon Western Chemiluminescent HRP Substrate (Millipore, Burlington, MA, USA) and visualized using a ChemiDoc Imager (Bio-Rad, Hercules, CA, USA) with Image Lab software (Bio-Rad, Hercules, CA, USA). PKG was probed as loading control using a rabbit polyclonal human-PKG antibody (Enzo Lifesciences, Lörrach, Germany, diluted 1:1,000), followed by a HRP-conjugated goat anti-rabbit secondary antibody (Sigma, St. Louis, MO, USA, diluted 1:3,000).

### Immunofluorescence assays

For IFA of PF3D7_1252600-smMyc and PI-PLC-GFP-KS parasites, air-dried thin blood films were fixed for 3 minutes in icecold methanol. After rehydration in PBS and blocking in 3% BSA/PBS, they were stained in blocking buffer with the following primary antibodies: rabbit anti-Myc (Cell Signalling Technology, Danvers, MA, USA, #2272, 1:1,000), mouse anti-AMA1 antibody 1F9 (59) (1:1,000), and mouse monoclonal anti-RAP1 antibody 2.29 (60) (1:1,000). This was followed by staining with the following secondary antibodies in blocking buffer additionally containing 1 µg/mL DAPI: anti-rabbit-AlexaFluor488 antibody (Invitrogen, Waltham, MA, USA, 1:2,000), anti-mouse-AlexaFluor594 antibody (Invitrogen, Waltham, MA, USA, 1:2,000), and anti-mouse-AlexaFluor488 antibody (Invitrogen, Waltham, MA, USA, 1:2,000). Finally, DAKO-mounting solution was added, and slides were covered with a coverslip.

For IFA of PI-PLC:HA:loxPint parasites, air-dried thin blood films were fixed with 4% paraformaldehyde in PBS for 30 minutes at room temperature (RT), permeabilized with 0.1% (vol/vol) Triton X-100 in PBS for 10 minutes, and blocked overnight in 4% BSA/PBS. Samples were probed with rat anti-HA 3F10 (Sigma, St. Louis, MO, USA, 1:500) in 4% BSA/PBS. Bound primary antibodies were detected using biotin-conjugated anti-rat antibody (Roche, Basel, Switzerland, 1:1,000) and AlexaFluor594-conjugated streptavidin (Life Technologies, Carlsbad, CA, USA, 1:1,000) in 4% BSA/PBS. Slides were mounted in ProLong Gold Antifade Mountant with DAPI (Life Technologies, Carlsbad, CA, USA).

### Analysis of SLI-based parasite lines

Schizont-stage parasites of all analyzed parasite lines were isolated by Percoll enrichment and incubated with uninfected RBCs (5% hematocrit) for 3 hours to allow rupture and invasion. Parasites were then treated with 5% sorbitol to remove residual unruptured schizonts, leading to a synchronous ring-stage culture with a 3-hour window.

For growth analysis of TGD-based KO lines, synchronous ring-stage cultures were allowed to mature to trophozoites for 1 day. Parasitemia was then determined 1 day post infection by flow cytometry and adjusted to exactly 0.1% starting parasitemia in a 2-mL dish. Medium was changed daily, and growth of the parasite lines was assessed by flow cytometry after 5 days (two erythrocytic cycles). As a reference, WT parasites were included in each assay.

For growth analysis of PF3D7_1252600-cKO and PI-PLC-GFP-KS parasites, synchronous ring-stage cultures were adjusted to ~0.1% parasitemia and divided into two 2-mL dishes. To one of these dishes, rapalog (AP21967, Clontech, San Jose, CA, USA) was added to a final concentration of 250 nM (rapalog was stored at −20°C as a 500-mM stock in ethanol, and working stocks were kept as 1:20 dilutions in RPMI at 4°C), while the other dish served as a control. Parasitemia was analyzed by flow cytometry after 1, 3, 5, and 7 days when most of the parasites were at the trophozoite stage. After analysis on day 5, cultures were diluted 10-fold into fresh RBCs to prevent overgrowth. Medium with or without rapalog was changed daily.

For quantification of developmental stage and schizont analysis of PI-PLC-GFP-KS parasites, synchronous ring-stage cultures were diluted to ~1%–2% parasitemia in 2-mL dishes, which were either left untreated or treated with rapalog as described above. Giemsa-stained blood films were prepared at 24, 40, and 48 hpi. For stage quantification, at least 20 fields of view were recorded using a 63× objective per sample. Erythrocyte numbers were then determined using the automated Parasitemia software (http://www.gburri.org/parasitemia/), and the number of the different parasite stages was manually counted on these images. For analysis of schizont morphology, cultures containing schizont-stage parasites (40 hpi) were supplemented with the egress inhibitor compound 2 (1 μM; kindly provided by S. Osborne (LifeArc, London, UK) and stored as a 10-mM stock in DMSO at −20°C). After 8 hours, Giemsa-stained blood films were prepared, and schizont morphology was investigated by light microscopy.

### Analysis of PF3D7_1476800 and PI-PLC cKO parasites

Tightly synchronized ring-stage cultures were divided into two dishes and treated with 100 nM rapamycin (Sigma, St. Louis, MO, USA, prepared as a 10-mM stock in DMSO) or DMSO only for 3 hours at 37°C, following which the cultures received fresh medium. Twenty-four hours later, growth assays were set up for each treatment. For this, trophozoite-stage parasites were diluted in triplicate cultures with fresh RBCs to a parasitemia of 0.1%. Giemsa smears were prepared at selected time points, and parasite development and morphology were assessed and quantified by light microscopy. In order to enrich the cultures with mature schizont-stage parasites, parasites were treated at 46 hpi for 3 hours with 1 µM C2 to arrest egress.

### Flow cytometry

For growth quantification of PF3D7_1476800 and PI-PLC cKO parasite lines, parasites were fixed with 0.1% glutaraldehyde/PBS and stained with SYBR Green I dye (1:10,000 dilution in PBS; Life Technologies, Carlsbad, CA, USA) for 30 minutes at 37°C. Samples were analyzed in a BD Fortessa FACS instrument using the 530/30-blue detector configuration. Flow cytometry data were analyzed using FlowJo v10. Erythrocytes were gated based on their forward and side scatter parameters, and SYBR Green I stain-positive RBCs were identified using the 530/30-blue detector.

Flow cytometry-based analysis of growth of all other parasite lines was performed essentially as described previously (61). In brief, 20 µL resuspended parasite culture was incubated with dihydroethidium (5 µg/mL, Cayman Chemical, Ann Arbor, MI, USA) and SYBR Green I dye (0.25× dilution, Invitrogen, Waltham, MA, USA) in a final volume of 100 µL medium for 20 minutes at RT protected from light. Samples were analyzed on an ACEA NovoCyte flow cytometer. RBCs were gated based on their forward and side scatter parameters. For every sample, 100,000 events were recorded, and parasitemia was determined based on SYBR Green I fluorescence.

### Transmission electron microscopy

PI-PLC:HA:loxPint parasites were treated at ring stage with RAP or DMSO, as described above, and allowed to develop to schizont stage. Schizonts were Percoll enriched and incubated with 1 µM C2 for 3 hours. Samples were then fixed with 2.5% glutaraldehyde-4% formaldehyde in 0.1 M phosphate buffer (PB) for 30 minutes at RT.

Schizonts were embedded in 3% low melting point agarose, and the samples then cut into 1 mm^3^ blocks. These were then processed using a modified version of the NCMIR protocol (62). Briefly, blocks were washed in 0.1 M PB, post-fixed with 1% reduced osmium [1% OsO_4_/1.5% K_3_Fe(CN)_6_] for 1 hour at 4°C, then washed in double distilled water (ddH_2_O). The blocks were incubated in 1% thiocarbohydrazide for 20 minutes at RT, rinsed in ddH_2_O, and further fixed with 2% osmium tetroxide for 30 minutes at RT. The blocks were then stained with 1% uranyl acetate at 4°C overnight, washed in ddH_2_O, and stained with Walton’s lead aspartate for 30 minutes at 60°C. The blocks were washed in ddH_2_O and dehydrated stepwise using serial dilutions of ethanol: 30% and 50% at RT for 5 minutes each and then 70%, 90%, and 2 × 100% for 10 minutes each. The blocks were infiltrated with a 4:1 mixture of propylene oxide (PO):Durcupan resin (Sigma, St. Louis, MO, USA) for 1 hour at RT, followed by 1:1 and 1:4 mixtures for 1 hour each at RT and then with 100% Durcupan resin for 48 hours. Blocks were polymerized in fresh Durcupan resin at 60°C for 48 hours. The samples were cut into 70 nm ultrathin sections using an ultramicrotome (UC7, Leica Microsystems, UK) and picked up onto copper mesh grids (Agar Scientific, Stansted, UK). Images were obtained on a 120-kV transmission electron microscope (Tecnai G2 Spirit BioTwin, Thermo Fisher Scientific, Waltham, MA, USA) using a charge-coupled device camera (Oneview, Gatan Inc., Pleasanton, CA, USA).

### Lipidomic analysis and PIP_x_ quantitation

Highly synchronous PI-PLC-GFP-KS ring-stage parasite cultures were divided into eight 10-mL plates. Four of these were treated with 250 nM rapalog, while the other four were left untreated. Medium with or without rapalog was replaced once per day. At 30 and 40 hpi, parasitemia (9%–15%) and the total number of erythrocytes per milliliter of parasite culture were determined by flow cytometry for calculation of absolute parasite numbers. Per treatment and time point, parasites from two 10-mL dishes were isolated by saponin lysis (corresponding to 379–647 × 10^6^ parasites per sample). For this, infected erythrocytes were first washed in icecold PBS, followed by incubation in 0.03% saponin in PBS on ice for 10 minutes. After three washes in icecold PBS, parasite pellets were stored at −80°C until lipid extraction.

Parasite pellets were suspended with water to achieve standardized cell numbers for lipidomics and PIP_x_ analysis. Samples were afterward homogenized using three freeze/thaw cycles using liquid nitrogen and sonication. Directly afterward, aliquots of the homogenate were transferred into a new vial for lipidomics corresponding to 25 × 10^6^ cells and a vial for PIP_x_ analysis corresponding to 200 × 10^6^ cells.

For lipidomics, a mix of internal standards was added (Supplemental file 3), and lipid extraction was performed according to an earlier described lipid extraction method using methyl-tert-butyl ether (MTBE) (63). Cholesterol was determined after acetylation as described (64). Shotgun lipidomics measurements were performed as described earlier (65) using Q Exactive Plus (Thermo Fisher Scientific, Bremen, Germany) mass spectrometer coupled with the TriVersa NanoMate (Advion, Ithaca, NY, USA). Lipid identification was performed with LipidXplorer 1.2.8 (66), and post processing including quantitation was executed with lxPostman. Total protein content was determined from the dried pellet and water phase after MTBE extraction using bicinchoninic acid (BCA) assay (Thermo Fisher Scientific, Waltham, MA, USA). Pellets were dissolved in 150-µL sample buffer, mixed well, and sonicated for 2 minutes. After a short centrifugation step, the samples were incubated for 30 minutes at 99°C, shaked at 1,400 rpm (Thermomixer, Eppendorf, Hamburg, Germany), and sonicated every 5 minutes for 30 seconds. After cooling down to 25°C, the assay was carried out according to the manufacturer’s instructions. Finally, absorbance was measured at 562 nm using infinite M200 Pro plate reader (Tecan, Männedorf, Switzerland).

For PIP_x_ analysis, the method of Clark et al. (50) was modified in respect of used internal standards (Supplemental file 3) and permethylation with diazomethane. Briefly, the organic phase was dried in a speedvac and afterward dissolved in 100-µL chloroform/methanol (95/5, vol/vol). To each sample, 60 µL diazomethane was added and incubated under a slight stream of nitrogen until the solution got clear. Two further additions of 60 µL diazomethane were performed with incubations of 5 and 15 minutes at room temperature. Finally, the samples were dried under a slight stream of nitrogen and afterward dissolved in 100 µL ACN (0.1% FA). LC-MS/MS was performed using a Xevo TQ (Waters, Manchester, UK) coupled with an 1100/1200 LC system (Agilent, Waldbronn, Germany). LC analysis was performed with a Jupiter C4 column (50 × 2 mm, 5 µm particle size) using a gradient of solvent A (water with 0.1% FA) and solvent B (ACN with 0.1% FA) at a flow rate of 500 µL/min and injection volume of 10 µL. Details on the LC Gradient and MRM transitions are listed in Supplemental file 3. Each sample was injected twice with first set of MRM transitions for internal standard and endogenous species with DAG backbone 38:4 and second acquisition consisting of transitions for DAG backbone 34:1 and 36:2.

### Statistical analysis

For statistical analysis of differences between two groups, paired or unpaired two-tailed Student’s *t*-tests were used. For statistical analysis of differences between more than two groups, a one-way analysis of variance, followed by a Holm-Sidak multiple comparison test, was performed. All statistical tests were done in GraphPad Prism. *P* values of <0.05 were considered significant. Statistical details (*n* numbers, tests used, definition of the error bars) are described in the figure legends.

## Data Availability

All data generated or analyzed during this study are included in this published article and its supplemental material files. Detailed information on the lipidomics approach is available on the LIFS webportal (68). The LipidCompass accession number is LCE00000008.

## SUPPLEMENTAL MATERIAL

FIG. S1 mbio.01413-23-s0001.tif Putative phospholipases of *P. falciparum*. RNAseq expression data are derived from reference 22. Orthologues in the rodent malaria model *P. berghei* are shown and non-syntenic ones are marked with an asterisk. Orthologues were identified in PlasmoDB (7) and are based on (67). Results of the genome-wide KO screens in *P. berghei* (plasmoGEM) (21) and *P. falciparum* using piggyBac-based mutagenesis (20) are shown. SP, signal peptide; TM, transmembrane domain; MS, Mass-spectrometry expression data; n.d./n.h., not determined/no homologue in *P. berghei*. Note that the putative lysophopholipase PF3D7_0102400 was not included into this list as it is annotated as pseudogene (PlasmoDB) (7).

FIG. S2 mbio.01413-23-s0002.tif Integration PCRs and growth analysis of parasite lines derived from the TGD-based gene-deletion screen. (A) Agarose gel electrophoresis of PCR products amplified from genomic DNA of the indicated parasite lines as well as unmodified WT parasites. Primers used are as indicated in Fig. 1, demonstrating a product across the 5' (primer 1+3) and 3' (primer 2+4) integration junction (indicated as 5' int and 3' int, respectively) as well as quantitative absence of the original locus (primer 1+2, “orig. loc.”). Absence of this band indicates that no WT parasites remained in the parasite population. KI, knock in cell line. Fragment length of the markers is indicated once (bottom right). (B) Flow cytometry-based growth analysis of synchronous phospholipase KO parasite lines after two erythrocytic cycles in comparison to WT parasites. Raw parasitemia values with means ± SD of four independent growth experiments per parasite line are shown. WT parasites were included in each independent assay as a reference. For statistical analysis of growth rates of the different parasite lines in comparison to WT parasites, a one-way analysis of variance (ANOVA) followed by a Holm-Sidak multiple comparison test was performed. All statistically significant differences are indicated (**P* < 0.05).

FIG. S3 mbio.01413-23-s0003.tif PF3D7_1252600 is dispensable for asexual blood-stage proliferation. (A) Schematic representation of the functional domain of PF3D7_1252600. (B) Schematic of the strategy used to endogenously tag PF3D7_1252600 with smMyc. T2A, skip peptide; Neo-R, neomycin-resistance gene; hDHFR, human dihydrofolate reductase. Localization of primers used to detect successful integration of targeting construct by PCR are indicated by arrows. (C) Agarose gel electrophoresis of PCR products amplified from genomic DNA of PF3D7_1252600-smMyc as well as unmodified WT parasites. KI, knock in cell line. (D) Western blot analysis of PF3D7_1252600-Sm-myc schizonts. GAPDH served as a loading control. (E, F) Colocalization analysis of PF3D7_1252600-SM-myc (green) and the microneme marker AMA1 (E) or the rhoptry marker RAP1 (F) in schizonts of transgenic KI parasites. As a control, the same staining was also performed in parental WT parasites. Marker proteins are displayed in magenta, while DAPI-stained nuclei are shown in blue. DIC, differential interference contrast. Scale bars, 5 μm. (G) Schematic of the strategy used to generate a cKO of PF3D7_1252600. HA, triple hemagglutinin tag; loxPint, loxP site within artificial intron. Primers used for integration PCR (1-4) and excision PCR (Ex) are indicated by arrows. (H) Agarose gel electrophoresis of PCR products amplified from genomic DNA of PF3D7_1252600-cKO as well as unmodified WT parasites. (I) Confirmation of efficient gene excision by PCR. Samples were taken at 27 or 72 hours post-infection (hpi). Expected PCR amplicon sizes from non-excised and excised parasites are shown. Shown is one representative out of three independent experiments. (J) Western blot of untreated or Rapa-treated PF3D7_1252600-cKO schizonts showing successful Rapa-induced ablation of PF3D7_1252600-3xHA expression. Samples were taken at the end of the erythrocytic cycle of Rapa-treatment (Cycle 1) or the following one (Cycle 2). GAPDH served as a loading control. Shown is one representative of two independent experiments. (K) Replication of untreated control (solid line) and Rapa-treated (dashed line) PF3D7_1252600-cKO parasites over four erythrocytic cycles. Shown are means ± SD of three independent experiments. For statistical analysis and unpaired Student's t-test was performed (n.s., not significant).

FIG. S4 mbio.01413-23-s0004.tif PF3D7_1476800 is dispensable for blood stage replication. (A) Schematic representation of the functional domain of PF3D7_1476800. (B) Schematic of the strategy used to generate a cKO of PF3D7_ PF3D7_1476800. Primers used for integration PCR (1-4) and excision PCR (Ex) are indicated by arrows and the site of targeted Cas9-mediated double-stranded DNA break is indicated with scissors. HR, homology region; HA, triple hemagglutinin tag; loxPint, loxP site within artificial intron; arrow, promoter. (C) Agarose gel electrophoresis of PCR products amplified from genomic DNA of PF3D7_1476800-cKO (clone C1) as well as unmodified WT parasites. (D) Confirmation of efficient gene excision by PCR. Samples were taken at 24 hours after RAP or mock (DMSO) treatment. Expected PCR amplicon sizes from non-excised and excised parasites are shown. Shown is one representative out of three independent experiments. (E) Replication of DMSO-treated (solid line) and RAP-treated (dashed line) PF3D7_1476800-cKO parasites over four erythrocytic cycles. Shown are means ± SD of three independent experiments.

FIG. S5 mbio.01413-23-s0005.tif Endogenous tagging of PI-PLC with 2xFKBP-GFP-2xFKBP. (A) Schematic of the strategy used for generation of PI-PLC-GFP-KS parasites. Localization of primers used to detect successful integration of targeting construct by PCR are indicated. 2A, skip peptide; hDHFR, human dihydrofolate reductase; Neo-R, neomycin-resistance gene; asterisks, stop codons. (B) Agarose gel electrophoresis of PCR products amplified from genomic DNA of PI-PLC-GFP-KS as well as unmodified WT parasites. KI, knock in cell line.

FIG. S6 mbio.01413-23-s0006.tif Integration PCR of PI-PLC:HA:loxPint parasites. Schematic of the pi-plc locus before and after CRISPR-Cas9-based gene editing is shown on top, while agarose gel electrophoresis of PCR products from unmodified WT and clonal modified parasite lines are displayed below. Primers used for confirming correct integration into the genome are indicated with half arrows.

FIG. S7 mbio.01413-23-s0007.tif Lipidomic analysis of untreated and Rapa-treated PI-PLC-GFP-KS parasites. Synchronous parasites were grown in absence (control) or presence of Rapa and harvested at 30 and 40 hpi. After releasing parasites from their host cell using saponin treatment, lipids were isolated and subjected to lipidomic analysis. The abundance of lipids was quantified and normalized to the amount of protein that was determined in parallel. Lipid levels in untreated control parasites at 30 and 40 hpi is shown in panel A, while lipid levels in control and Rapa-treated parasites at 30 and 40 hpi is displayed in panel B. Data are based on 8 biological replicates per treatment. Means ± SD are shown. Statistical significance was assessed with paired Student's t-test. All statistically significant differences are indicated (**P* < 0.05; ***P* < 0.01; ****P* < 0.001; *****P* < 0.0001). PC, phosphatidylcholine; PCO, alkyl-acylglycerophosphocholines; PE, phosphatidylethanolamine; PEO, alkyl-acylglycerophosphoethanolamines; PI, phosphatidylinositol; PS, phosphatidylserine; SM, sphingomyelin; TAG, triacylglycerol; DAG, diacylglycerol; FC, free cholesterol; PA, phosphatidic acid; PG, phosphatidylglycerol; LPC, lysophosphatidylcholine; LPE, lysophosphatidylethanolamine; Cer, ceramide. For a complete overview of all the results of the lipidomic analyses, please see Supplemental File 2.

Supplemental File 1 mbio.01413-23-s0008.xlsx Oligonucleotides and other synthetic DNA used in this study.

Supplemental File 2 mbio.01413-23-s0009.xlsx Lipidomic analysis of untreated and Rapa-treated PI-PLC-GFP-KS parasites. Source data for Fig. 4 and Fig. S7.

Supplemental File 3 mbio.01413-23-s0010.xlsx Method details on lipidomic analysis and PIPx quantitation.

ASM does not own the copyrights to Supplemental Material that may be linked to, or accessed through, an article. The authors have granted ASM a non-exclusive, world-wide license to publish the Supplemental Material files. Please contact the corresponding author directly for reuse.
