# Supplementary figures and images for: Global analysis of putative phospholipases in Plasmodium falciparum reveals an essential role of the phosphoinositide-specific phospholipase C in parasite maturation
Source: mBio. 2023 Jul 25;14(4):e01413-23. doi: 10.1128/mbio.01413-23 (PMC10470789; doi:10.1128/mbio.01413-23)

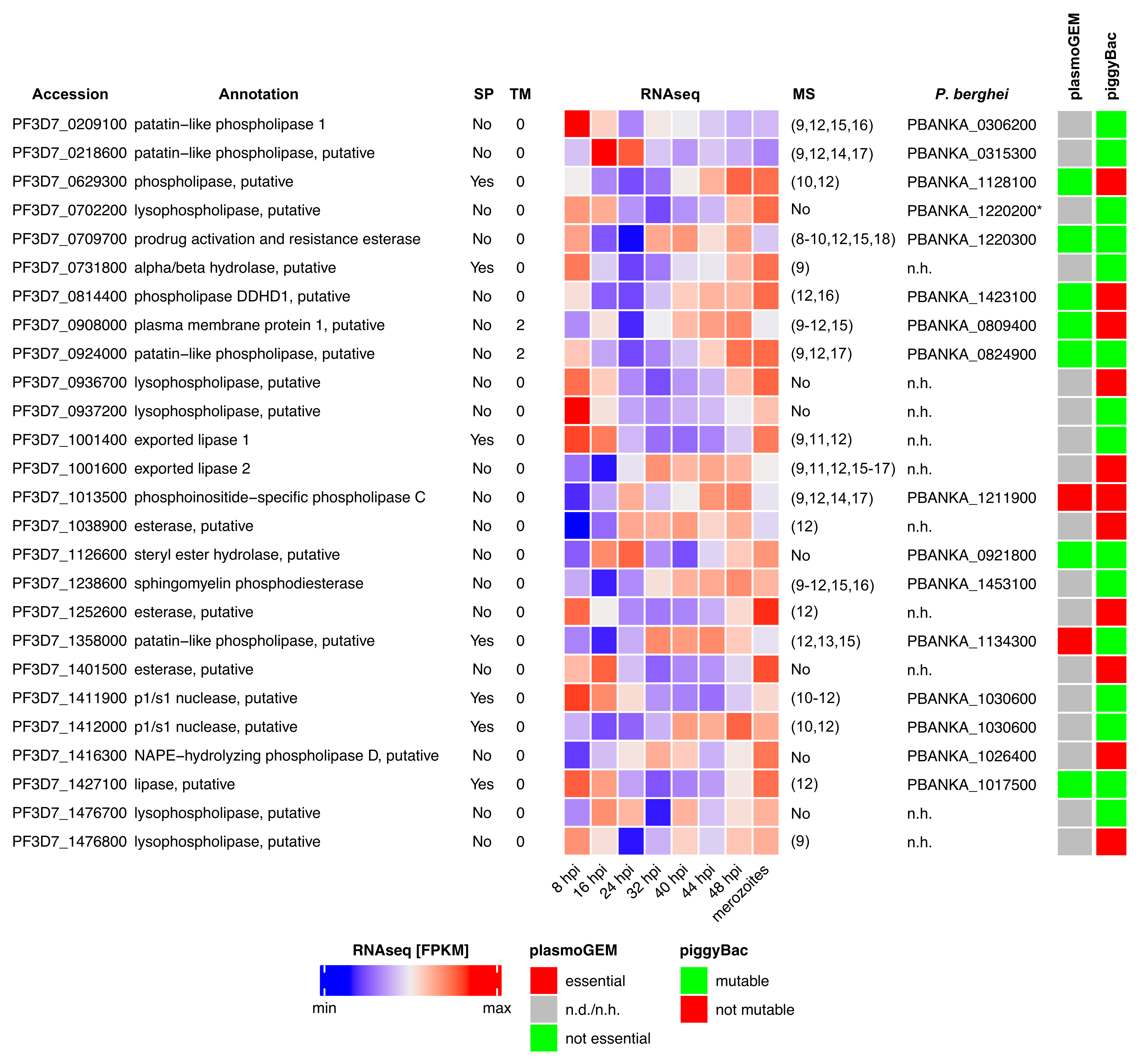

Supplement: FIG. S1 — Putative phospholipases of P. falciparum. RNAseq expression data are derived from reference 22. Orthologues in the rodent malaria model P. berghei are shown and non-syntenic ones are marked with an asterisk. Orthologues were identified in PlasmoDB (7) and are based on (67). Results of the genome-wide KO screens in P. berghei (plasmoGEM) (21) and P. falciparum using piggyBac-based mutagenesis (20) are shown. SP, signal peptide; TM, transmembrane domain; MS, Mass-spectrometry expression data; n.d./n.h., not determined/no homologue in P. berghei. Note that the putative lysophopholipase PF3D7_0102400 was not included into this list as it is annotated as pseudogene (PlasmoDB) (7). [file mbio.01413-23-s0001.tif]

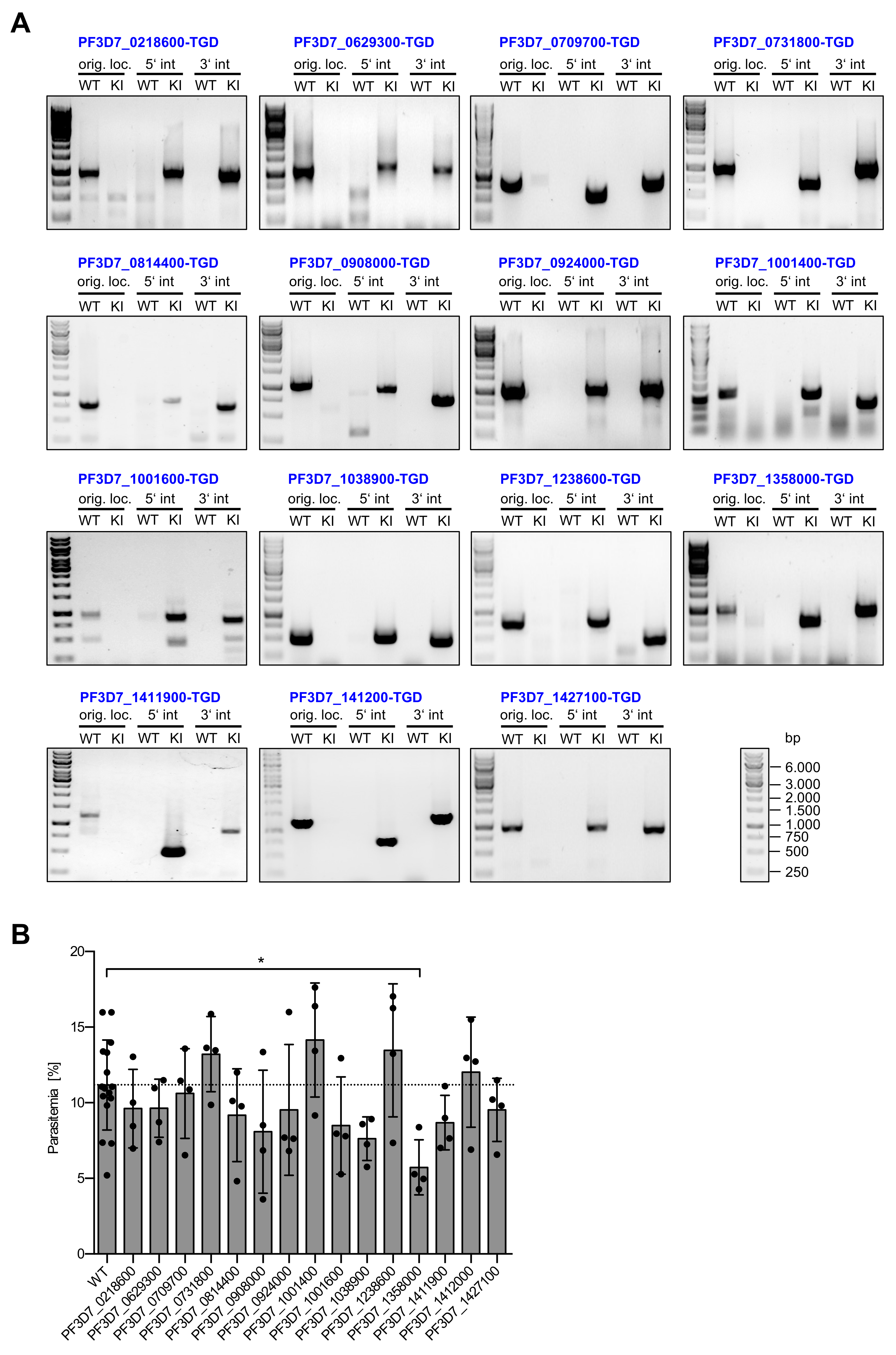

Supplement: FIG. S2 — Integration PCRs and growth analysis of parasite lines derived from the TGD-based gene-deletion screen. (A) Agarose gel electrophoresis of PCR products amplified from genomic DNA of the indicated parasite lines as well as unmodified WT parasites. Primers used are as indicated in Fig. 1, demonstrating a product across the 5' (primer 1+3) and 3' (primer 2+4) integration junction (indicated as 5' int and 3' int, respectively) as well as quantitative absence of the original locus (primer 1+2, “orig. loc.”). Absence of this band indicates that no WT parasites remained in the parasite population. KI, knock in cell line. Fragment length of the markers is indicated once (bottom right). (B) Flow cytometry-based growth analysis of synchronous phospholipase KO parasite lines after two erythrocytic cycles in comparison to WT parasites. Raw parasitemia values with means ± SD of four independent growth experiments per parasite line are shown. WT parasites were included in each independent assay as a reference. For statistical analysis of growth rates of the different parasite lines in comparison to WT parasites, a one-way analysis of variance (ANOVA) followed by a Holm-Sidak multiple comparison test was performed. All statistically significant differences are indicated (*P < 0.05). [file mbio.01413-23-s0002.tif]

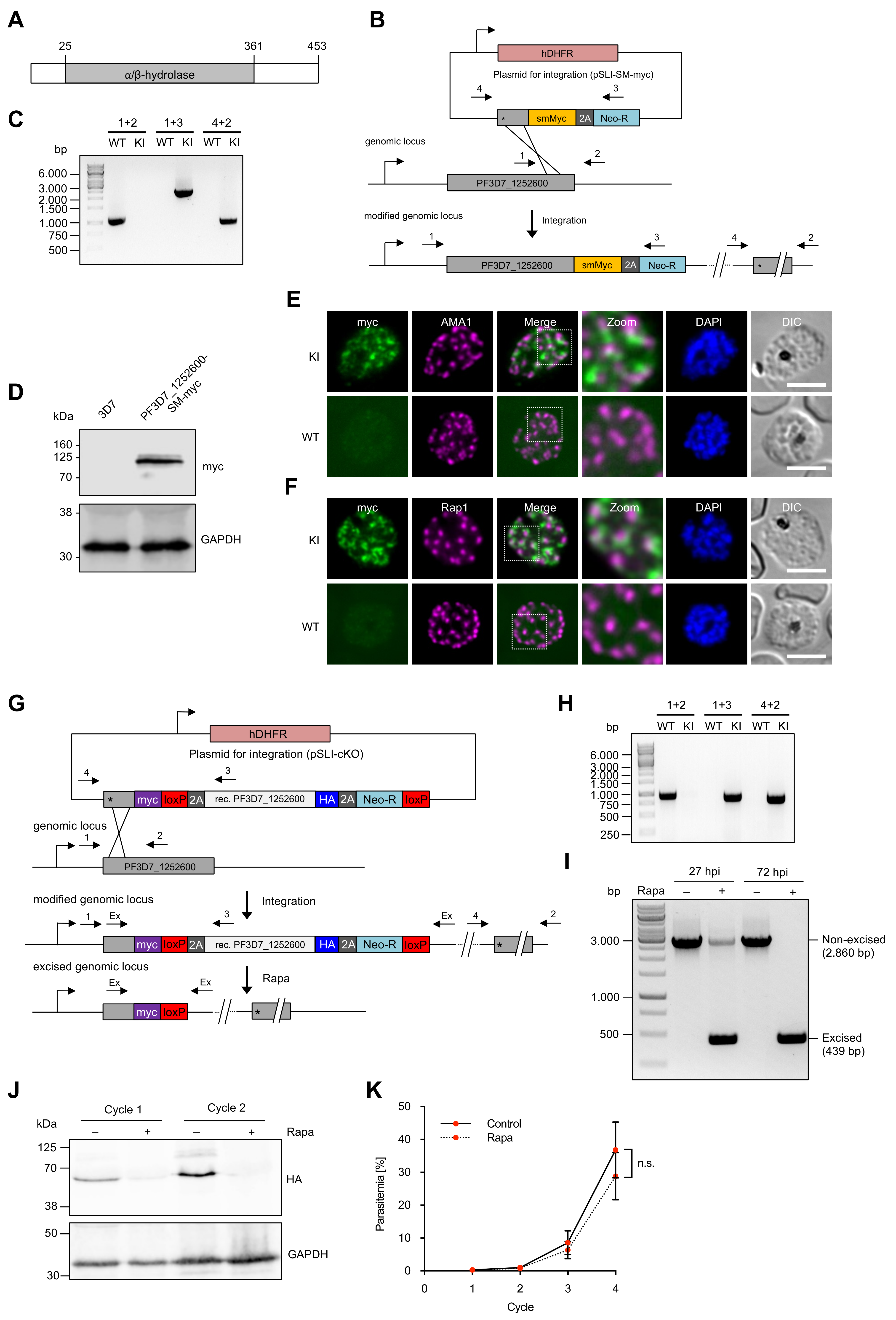

Supplement: FIG. S3 — PF3D7_1252600 is dispensable for asexual blood-stage proliferation. (A) Schematic representation of the functional domain of PF3D7_1252600. (B) Schematic of the strategy used to endogenously tag PF3D7_1252600 with smMyc. T2A, skip peptide; Neo-R, neomycin-resistance gene; hDHFR, human dihydrofolate reductase. Localization of primers used to detect successful integration of targeting construct by PCR are indicated by arrows. (C) Agarose gel electrophoresis of PCR products amplified from genomic DNA of PF3D7_1252600-smMyc as well as unmodified WT parasites. KI, knock in cell line. (D) Western blot analysis of PF3D7_1252600-Sm-myc schizonts. GAPDH served as a loading control. (E, F) Colocalization analysis of PF3D7_1252600-SM-myc (green) and the microneme marker AMA1 (E) or the rhoptry marker RAP1 (F) in schizonts of transgenic KI parasites. As a control, the same staining was also performed in parental WT parasites. Marker proteins are displayed in magenta, while DAPI-stained nuclei are shown in blue. DIC, differential interference contrast. Scale bars, 5 μm. (G) Schematic of the strategy used to generate a cKO of PF3D7_1252600. HA, triple hemagglutinin tag; loxPint, loxP site within artificial intron. Primers used for integration PCR (1-4) and excision PCR (Ex) are indicated by arrows. (H) Agarose gel electrophoresis of PCR products amplified from genomic DNA of PF3D7_1252600-cKO as well as unmodified WT parasites. (I) Confirmation of efficient gene excision by PCR. Samples were taken at 27 or 72 hours post-infection (hpi). Expected PCR amplicon sizes from non-excised and excised parasites are shown. Shown is one representative out of three independent experiments. (J) Western blot of untreated or Rapa-treated PF3D7_1252600-cKO schizonts showing successful Rapa-induced ablation of PF3D7_1252600-3xHA expression. Samples were taken at the end of the erythrocytic cycle of Rapa-treatment (Cycle 1) or the following one (Cycle 2). GAPDH served as a loading control. Shown i [file mbio.01413-23-s0003.tif]

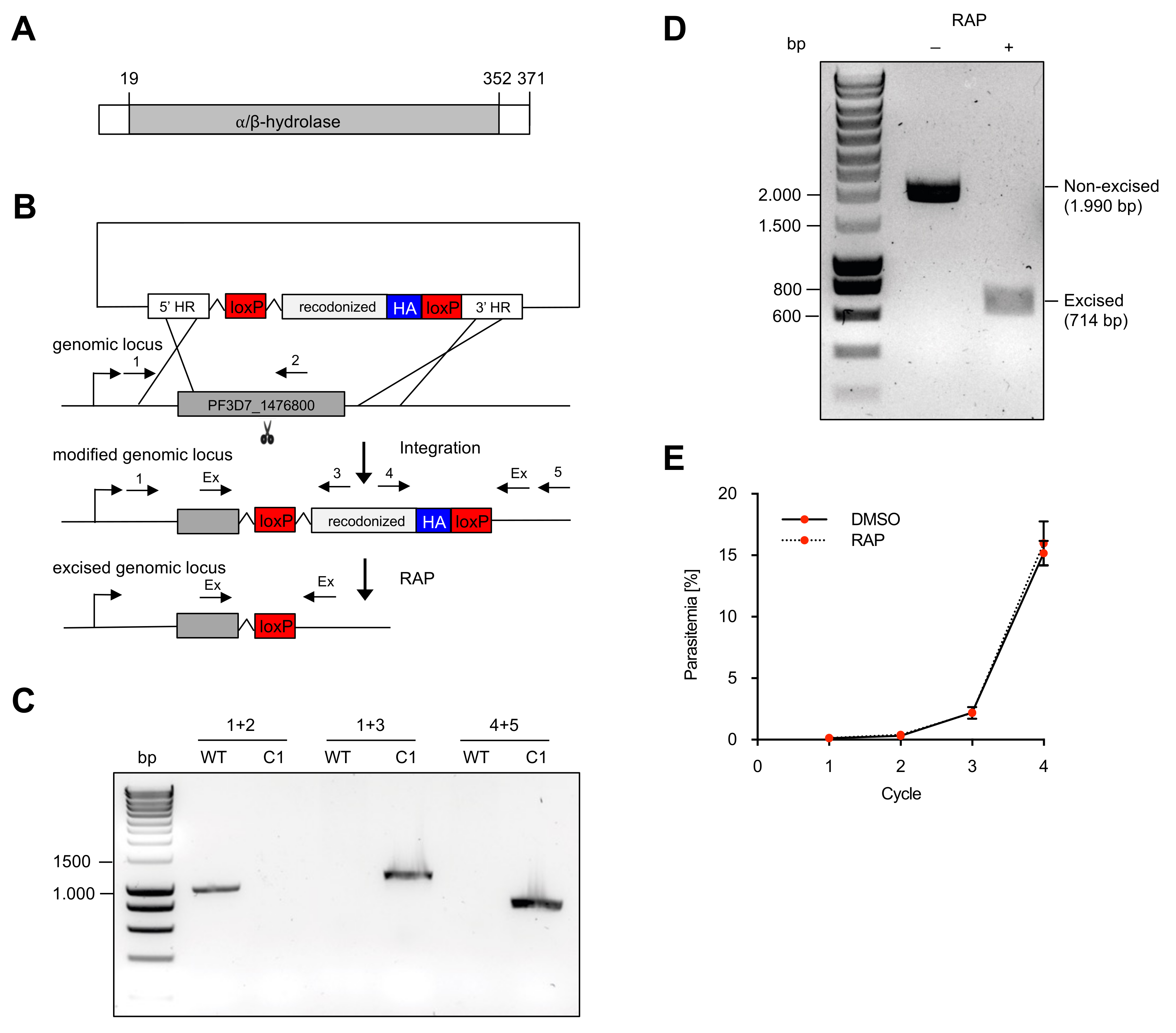

Supplement: FIG. S4 — PF3D7_1476800 is dispensable for blood stage replication. (A) Schematic representation of the functional domain of PF3D7_1476800. (B) Schematic of the strategy used to generate a cKO of PF3D7_ PF3D7_1476800. Primers used for integration PCR (1-4) and excision PCR (Ex) are indicated by arrows and the site of targeted Cas9-mediated double-stranded DNA break is indicated with scissors. HR, homology region; HA, triple hemagglutinin tag; loxPint, loxP site within artificial intron; arrow, promoter. (C) Agarose gel electrophoresis of PCR products amplified from genomic DNA of PF3D7_1476800-cKO (clone C1) as well as unmodified WT parasites. (D) Confirmation of efficient gene excision by PCR. Samples were taken at 24 hours after RAP or mock (DMSO) treatment. Expected PCR amplicon sizes from non-excised and excised parasites are shown. Shown is one representative out of three independent experiments. (E) Replication of DMSO-treated (solid line) and RAP-treated (dashed line) PF3D7_1476800-cKO parasites over four erythrocytic cycles. Shown are means ± SD of three independent experiments. [file mbio.01413-23-s0004.tif]

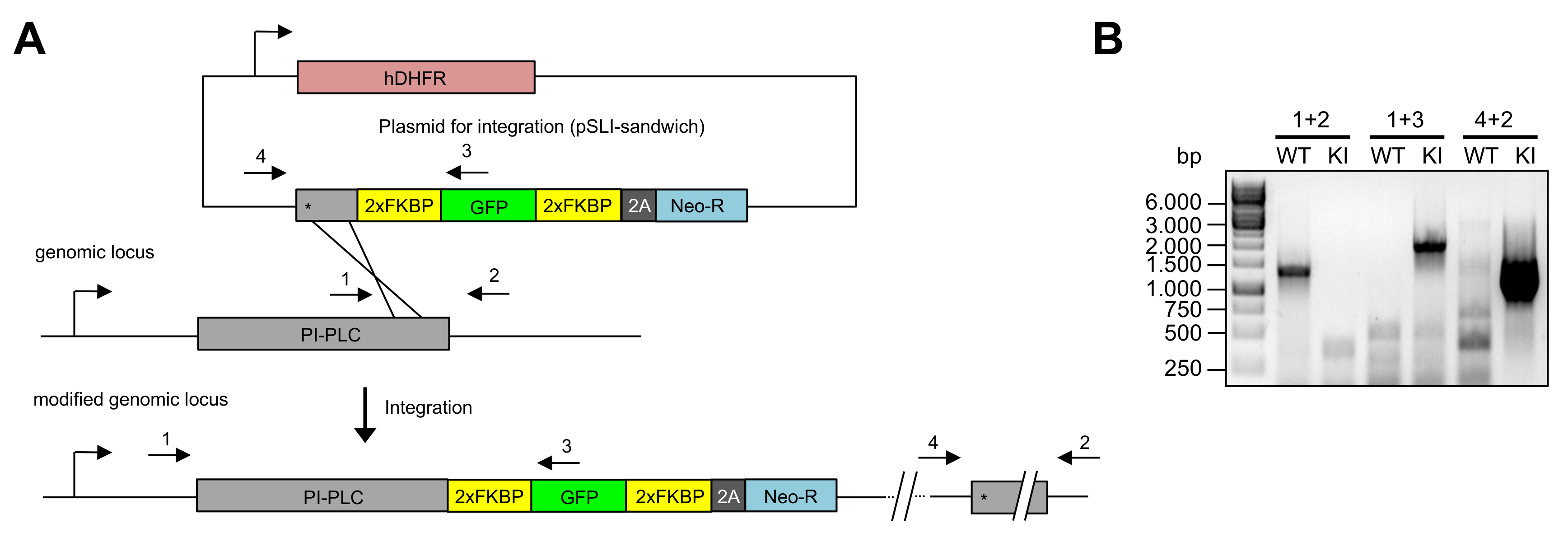

Supplement: FIG. S5 — Endogenous tagging of PI-PLC with 2xFKBP-GFP-2xFKBP. (A) Schematic of the strategy used for generation of PI-PLC-GFP-KS parasites. Localization of primers used to detect successful integration of targeting construct by PCR are indicated. 2A, skip peptide; hDHFR, human dihydrofolate reductase; Neo-R, neomycin-resistance gene; asterisks, stop codons. (B) Agarose gel electrophoresis of PCR products amplified from genomic DNA of PI-PLC-GFP-KS as well as unmodified WT parasites. KI, knock in cell line. [file mbio.01413-23-s0005.tif]

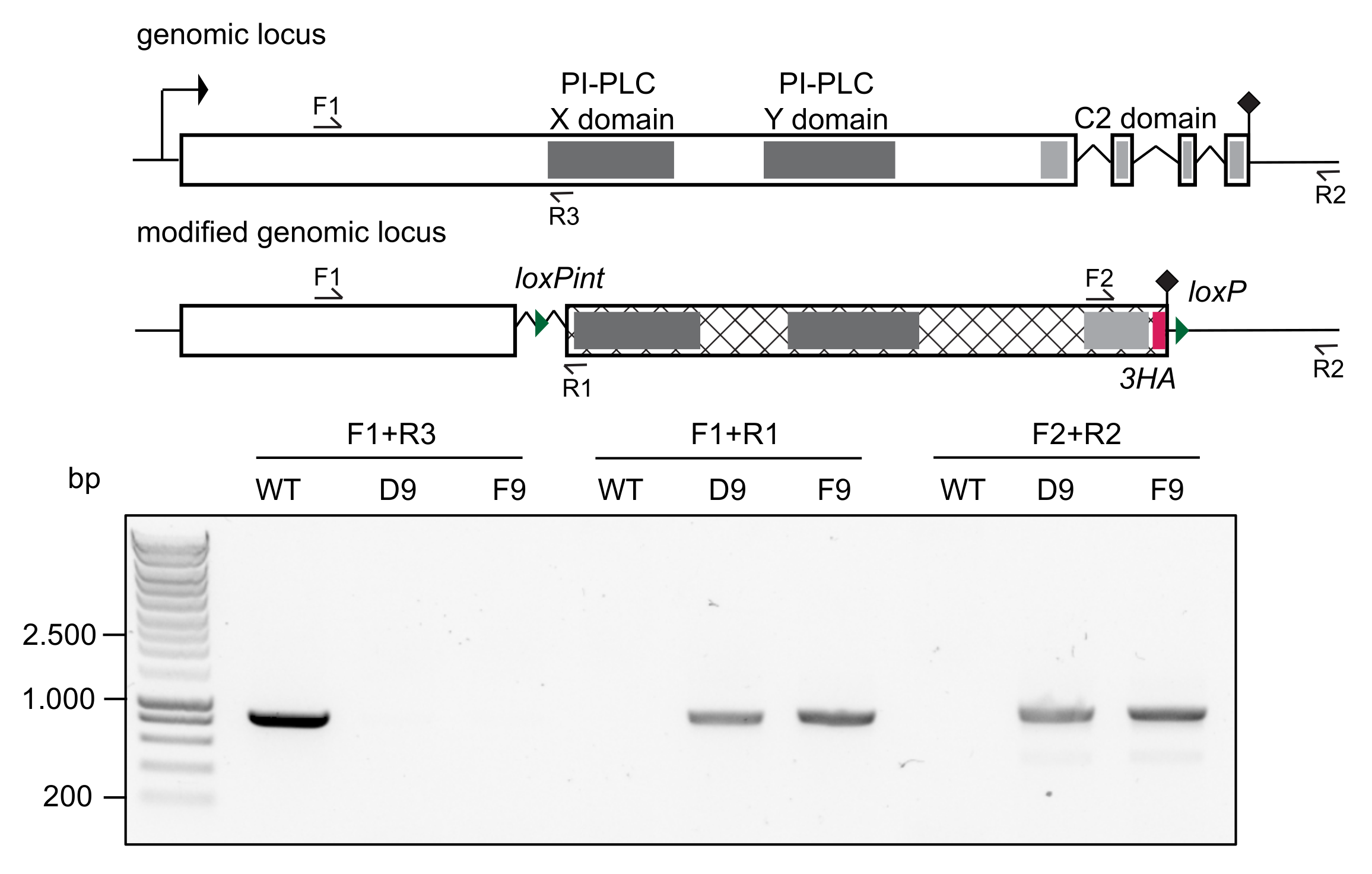

Supplement: FIG. S6 — Integration PCR of PI-PLC:HA:loxPint parasites. Schematic of the pi-plc locus before and after CRISPR-Cas9-based gene editing is shown on top, while agarose gel electrophoresis of PCR products from unmodified WT and clonal modified parasite lines are displayed below. Primers used for confirming correct integration into the genome are indicated with half arrows. [file mbio.01413-23-s0006.tif]

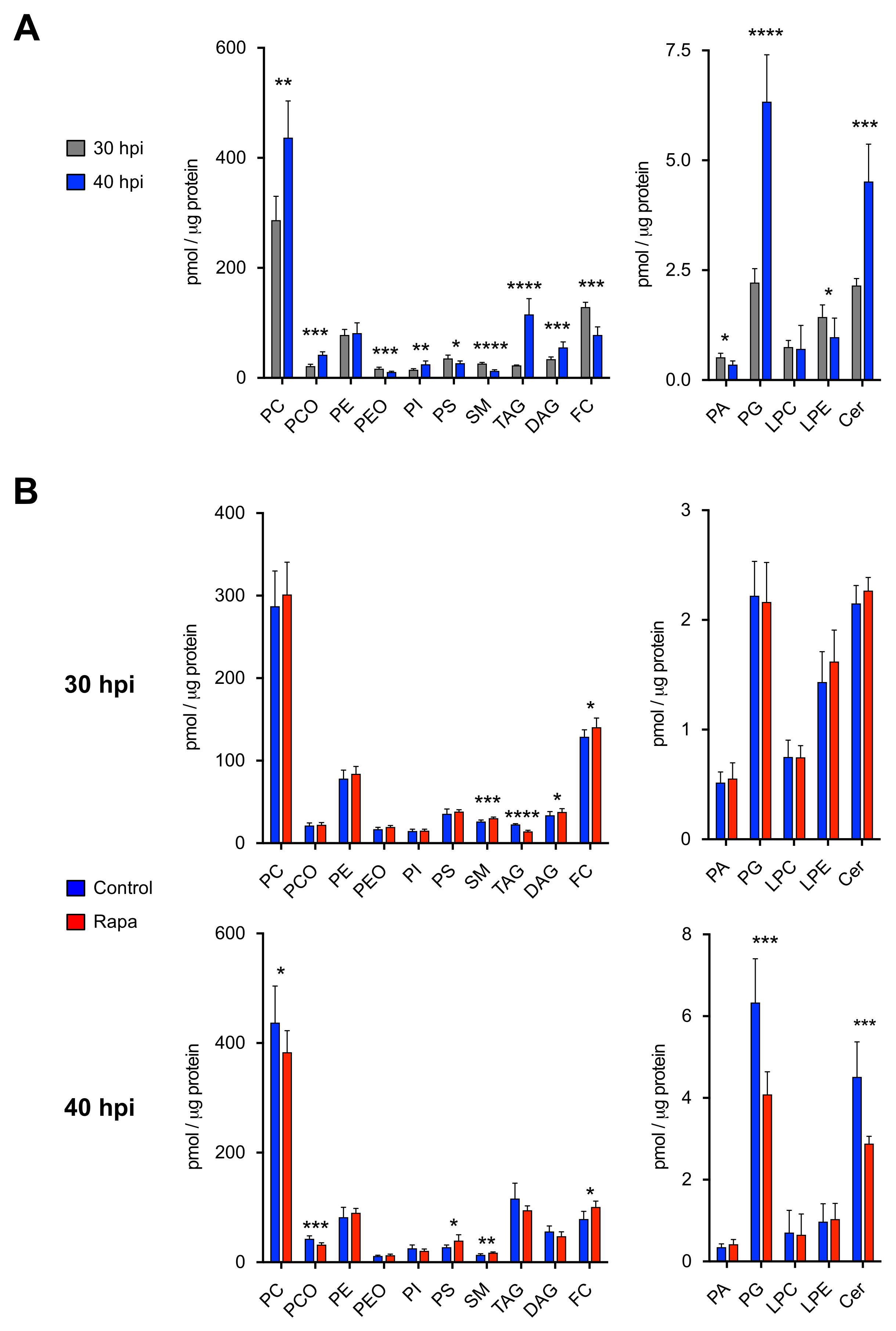

Supplement: FIG. S7 — Lipidomic analysis of untreated and Rapa-treated PI-PLC-GFP-KS parasites. Synchronous parasites were grown in absence (control) or presence of Rapa and harvested at 30 and 40 hpi. After releasing parasites from their host cell using saponin treatment, lipids were isolated and subjected to lipidomic analysis. The abundance of lipids was quantified and normalized to the amount of protein that was determined in parallel. Lipid levels in untreated control parasites at 30 and 40 hpi is shown in panel A, while lipid levels in control and Rapa-treated parasites at 30 and 40 hpi is displayed in panel B. Data are based on 8 biological replicates per treatment. Means ± SD are shown. Statistical significance was assessed with paired Student's t-test. All statistically significant differences are indicated (*P < 0.05; **P < 0.01; ***P < 0.001; ****P < 0.0001). PC, phosphatidylcholine; PCO, alkyl-acylglycerophosphocholines; PE, phosphatidylethanolamine; PEO, alkyl-acylglycerophosphoethanolamines; PI, phosphatidylinositol; PS, phosphatidylserine; SM, sphingomyelin; TAG, triacylglycerol; DAG, diacylglycerol; FC, free cholesterol; PA, phosphatidic acid; PG, phosphatidylglycerol; LPC, lysophosphatidylcholine; LPE, lysophosphatidylethanolamine; Cer, ceramide. For a complete overview of all the results of the lipidomic analyses, please see Supplemental File 2. [file mbio.01413-23-s0007.tif]
